# On Superposition Lattice Codes for the *K*-User Gaussian Interference Channel

**DOI:** 10.3390/e26070575

**Published:** 2024-07-03

**Authors:** María Constanza Estela, Claudio Valencia-Cordero

**Affiliations:** Department of Electrical Engineering, Universidad de Santiago de Chile (USACH), Santiago 9170022, Chile; claudio.valenciac@usach.cl

**Keywords:** interference channels, lattice Gaussian coding, flatness factor

## Abstract

In this study, we work with lattice Gaussian coding for a *K*-user Gaussian interference channel. Following the procedure of Etkin et al., in which the capacity is found to be within 1 bit/s/Hz of the capacity of a two-user Gaussian interference channel for each type of interference using random codes, we work with lattices to take advantage of their structure and potential for interference alignment. We mimic random codes using a Gaussian distribution over the lattice. Imposing constraints on the flatness factor of the lattices, the common and private message powers, and the channel coefficients, we find the conditions to obtain the same constant gap to the optimal rate for the two-user weak Gaussian interference channel and the generalized degrees of freedom as those obtained with random codes, as found by Etkin et al. Finally, we show how it is possible to extend these results to a *K*-user weak Gaussian interference channel using lattice alignment.

## 1. Introduction

Interference is one of the major issues of wireless communications. One important scenario corresponds to the interference channel, where each transmitter wishes to communicate with its correspondent receiver but, as all users share the wireless medium, there is interference between them. Interference is classified according to its level, from very strong to low. When interference is very strong, it has been demonstrated [1] that the capacity is the same as if there was no interference at all. This is because interference is decoded first. Interference is low when it falls below the level of noise. In this case, there is no loss of data rate due to interference. The problem is still open for moderate or weak interference. For this case, the conventional technique consists of orthogonalizing the signals using frequency or time division multiple access schemes. Interference alignment has been proposed from the scope of information theory to align interference at each receiver, using only half of the signal space and leaving the other half for the intended signal, independent of the number of users that the channel has. The sum capacity for the *K*-user interference channel has been characterized in [2], and it was found that at a high signal-to-noise ratio (SNR), a factor of K/2 dominates the capacity. This factor represents the degrees of freedom (DoF).

One of the main achievements in finding the capacity for a two-user interference channel can be seen in the work of Han and Kobayashi [3], who found the inner bound for the two-user interference channel using superposition coding. The method to determine the capacity of such an interference channel consists of using private and common messages from each transmitter. The private message of the interferer is treated as noise, while both common messages and the desired private message are decoded at each receiver. Obtaining similar results when *K* users are considered is desirable. It has been shown in [4] that, through using lattice codes, the interference due to one interferer can be made the same as that caused by many interferers. At each receiver, the signals can be scaled in such a way that the interference signals lie in a lattice, which can be distinguished from the lattice containing the desired signal. This was defined in [4] as lattice alignment. The signal scale idea has been studied in [5,6,7,8] to obtain the DoF of different interference channel models. In [5], a deterministic channel approach was applied to an interference channel, where signals are represented in base *Q*. In [9], the generalized degrees of freedom (GDoF) were found for different types of interference according to the SNR and interference-to-noise ratio (INR) for a two-user interference channel. Following the ideas of [5,9], in [6], the GDoF was found for different levels of interference for the *K*-user interference channel. The signals are represented in base *Q*, and a detailed scheme was given for different types of interference. New approaches have been made to find the GDoF for the *K*-user interference channel. In particular, in [10], the GDoF of a *K*-user interference channel was studied when treating interference as noise, which was found to be optimal depending on the relationship between the desired signal strength and the sum of the strengths of the strongest interference from and to this user. In [11], the GDoF of a *K*-user interference channel was studied using a multi-layer interference alignment scheme with successive decoding. The optimal sum of the GDoF was characterized by the exponents of each of the channel strengths.

Recently, interference alignment has been applied to different scenarios such as wireless interference channels for Smart Grids [12], unmanned aerial vehicles in heterogeneous networks [13] and space–air–ground integrated networks [14]. On the other hand, many of the lattice code techniques that are used in this paper have previously been considered for security. This is the case for [15,16], who worked with the secure capacity of wiretap channels, or [17], who worked with the secure DoF of the *K*-user interference channel. However, to the best of our knowledge, few researchers have recently studied the GDoF or constant gap to the optimal rate of the *K*-user interference channels using lattice alignment.

Following the ideas of [9], in [18], the GDoF of the two-user symmetric interference channel is found using a lattice Gaussian distribution. In this study, we propose extending these results for the *K*-user interference channel, using additive white Gaussian noise (AWGN)-good lattices. First, we begin with a two-user Gaussian interference channel, and work with lattice codes as we want to use the potential of lattices to align interference for a K-user Gaussian interference channel. For this purpose, we propose a lattice Gaussian coding scheme with some constraints over the powers of the messages and the flatness factor of the lattices. Using the intersection of two two-user multiple access channel rate regions, we find that we can achieve the conditions to obtain the same constant gap to the optimal rate and, thus, the same GDoF for a two-user weak interference channel, as found in [9], with lattice Gaussian codes. Finally, we show how to apply these results to a *K*-user interference channel using lattice alignment, with a careful selection of the lattices for each user.

### Roadmap

The remainder of this paper is organized as follows: In Section 2, the upper and inner bounds and the GDoF of the two-user interference channel obtained in [9] are shown, and important Lemmas and Theorems of the lattice Gaussian coding [19] are explained. The main results of this work are stated in Theorems 3 and 4 in Section 4, which identify the channel coefficient conditions to obtain the same GDoF as in [9]. To prove this, we perform the following:

- In Section 3.1.1, we show it is possible to obtain the HK rate region for a two-user interference channel with the intersection of two two-user multiple access channels.
- In Section 3.1.2, we express the HK rate region for a two-user Gaussian interference channel with lattice distribution (Section 3.1.2 for a *K*-user Gaussian interference channel). For this, we introduce restrictions over the flatness factor of lattices given by Lemmas 3 and 4, as well as Theorem 2.
- Finally, in Section 4.1, for Lemma 9, we apply power constraints to the private and common messages of a two-user weak Gaussian interference channel (Lemma 10 for a *K*-user weak Gaussian interference channel). These constraints are then applied to obtain conditions for the channel coefficients (Theorem 3 for a two-user weak Gaussian interference channel and Theorem 4 for a *K*-user weak Gaussian interference channel), which finally lead to the constant gap to the optimal rate and the GDoF of the two-user interference channel obtained in [9].

In Section 5, we discuss and highlight the results obtained. Finally, the conclusions of this work are drawn in Section 6.

## 2. Preliminaries

A study by Etkin et al. [9] revealed the capacity of the two-user interference channel within 1 Bit/s/Hz. When the power of the interference is smaller than the power of the desired signal, a range of values in which the Han and Kobayashi achievable rate (hereafter, the HK rate) is contained can be found. The GDoF is found through normalizing this rate by the capacity of the point-to-point AWGN channel and taking the limit of this ratio when the SNR and INR →∞. In order to do this, random Gaussian codes and a simple HK scheme are used. In this section, we show the results of [9] for a two-user weak interference channel and, later, we present the main results on lattice Gaussian coding [19], the Lemmas and Theorems of which are used for our later results.

### 2.1. Outer and Inner Bounds for the Two-User Weak Gaussian Interference Channel [9]

The channel model given in [9] is expressed as:

(1) $$\begin{array}{c} y_{i}=\sum_{j=1}^{2}h_{ji}x_{j}+z_{i}, \end{array}$$

where i,j=1,2, $x_{j}\in C$ are subject to a power constraint $E\left[|x_{j}{|}^{2}\right]=P_{j}$ and the noise is $z_{i}∼\mathrm{CN}\left(0,N_{0}\right)$. The channel coefficients from transmitter *i* to receiver *j* are represented by $h_{ji}$. Let ${\mathrm{SNR}}_{i}=\frac{|h_{ii}{|}^{2}P_{i}}{N_{0}}$ also be the SNR of user *i*, and ${\mathrm{INR}}_{1}=\frac{|h_{21}{|}^{2}P_{2}}{N_{0}}$ and ${\mathrm{INR}}_{2}=\frac{|h_{12}{|}^{2}P_{1}}{N_{0}}$. The authors in [9] provide a new outer bound for the two-user weak and mixed Gaussian interference channel. Here, we show their results for the weak interference case:

(2) $$\begin{array}{ccc} R_{1} & \leq & \log\left(1+{\mathrm{SNR}}_{1}\right) \end{array}$$

(3) $$\begin{array}{ccc} R_{2} & \leq & \log\left(1+{\mathrm{SNR}}_{2}\right) \end{array}$$

(4) $$\begin{array}{ccc} R_{1}+R_{2} & \leq & \log\left(1+{\mathrm{SNR}}_{2}\right)+\log\left(1+\frac{{\mathrm{SNR}}_{1}}{{\mathrm{INR}}_{1}+1}\right) \end{array}$$

(5) $$\begin{array}{ccc} R_{1}+R_{2} & \leq & \log\left(1+{\mathrm{SNR}}_{1}\right)+\log\left(1+\frac{{\mathrm{SNR}}_{2}}{{\mathrm{INR}}_{2}+1}\right) \end{array}$$

(6) $$\begin{array}{ccc} R_{1}+R_{2} & \leq & \log\left(1+{\mathrm{INR}}_{1}+\frac{{\mathrm{SNR}}_{1}}{{\mathrm{INR}}_{2}+1}\right)+\log\left(1+{\mathrm{INR}}_{2}+\frac{{\mathrm{SNR}}_{2}}{{\mathrm{INR}}_{1}+1}\right) \end{array}$$

(7) $$\begin{array}{ccc} 2R_{1}+R_{2} & \leq & \log\left(1+{\mathrm{SNR}}_{1}+{\mathrm{INR}}_{1}\right)+\log\left(1+{\mathrm{INR}}_{2}+\frac{{\mathrm{SNR}}_{2}}{{\mathrm{INR}}_{1}+1}\right)\\ & & +\log\left(\frac{1+{\mathrm{SNR}}_{1}}{1+{\mathrm{INR}}_{2}}\right) \end{array}$$

(8) $$\begin{array}{ccc} R_{1}+2R_{2} & \leq & \log\left(1+{\mathrm{SNR}}_{2}+{\mathrm{INR}}_{2}\right)+\log\left(1+{\mathrm{INR}}_{1}+\frac{{\mathrm{SNR}}_{1}}{{\mathrm{INR}}_{2}+1}\right)\\ & & +\log\left(\frac{1+{\mathrm{SNR}}_{2}}{1+{\mathrm{INR}}_{1}}\right). \end{array}$$

Later, as presented in [3], superposition coding is considered. The private message of user i=1,2 is represented as $u_{i}$, while the common message is represented as $w_{i}$. User *i* transmits the signal given by $x_{i}=u_{i}+w_{i}$. The private codeword $u_{i}$ is meant to be decoded only by user *i*, while it is treated as noise by the other user. Both $w_{1}$ and $w_{2}$ are decoded by both users. In [9], the codebooks are generated using i.i.d. random Gaussian variables, and the interference-to-noise ratio created by the private message is defined as ${\mathrm{INR}}_{p}$ and chosen as equal to 1. A simplified HK scheme is used in order to find the achievable region within 1 bit/s/Hz of the outer bound. To begin, in ([20] [Section 3.2]), a simplification of the HK rate region is found, which relies on the fact that many of the limits found in [3] are redundant. This has also been acknowledged by Han and Kobayashi in [21]. Consider the auxiliary variables given in [3], $U_{1},U_{2},W_{1},W_{2}$ and *Q*, where $U_{i}$ represents the private information from user *i*, $W_{i}$ represents the common information from user i=1,2, and *Q* is a time sharing parameter. Given the set $Z=(Q,U_{1},W_{1},U_{2},W_{2},X_{1},X_{2},Y_{1},Y_{2})$, the HK capacity rate region R(Z) is the set of all simultaneously achievable rate pairs $\left(R_{1},R_{2}\right)$ that satisfy ([20] [Section 3.2]):

(9) $$\begin{array}{ccc} R_{1} & \leq & \min\left\{I\left(Y_{1};W_{1}|W_{2}Q\right),I\left(Y_{2};W_{1}|U_{2}W_{2}Q\right)\right\}+I\left(Y_{1};U_{1}|W_{1}W_{2}Q\right), \end{array}$$

(10) $$\begin{array}{ccc} R_{2} & \leq & \min\left\{I\left(Y_{2};W_{2}|W_{1}Q\right),I\left(Y_{1};W_{2}|U_{1}W_{1}Q\right)\right\}+I\left(Y_{2};U_{2}|W_{1}W_{2}Q\right), \end{array}$$

(11) $$\begin{array}{ccc} R_{1}+R_{2} & \leq & \min\{I\left(Y_{1};W_{1}W_{2}Q\right),I\left(Y_{2};W_{1}W_{2}Q\right)\\ & & ,I\left(Y_{1};W_{2}|W_{1}Q\right)+I\left(Y_{2};W_{1}|W_{2}Q\right)\}\\ & & +I\left(Y_{1};U_{1}|W_{1}W_{2}Q\right)+I\left(Y_{2};U_{2}|W_{1}W_{2}Q\right), \end{array}$$

(12) $$\begin{array}{ccc} 2R_{1}+R_{2} & \leq & I\left(Y_{1};W_{1}W_{2}Q\right)+I\left(Y_{2};W_{1}|W_{2}Q\right)\\ & & +2I\left(Y_{1};U_{1}|W_{1}W_{2}Q\right)+I\left(Y_{2};U_{2}|W_{1}W_{2}Q\right) \end{array}$$

(13) $$\begin{array}{ccc} R_{1}+2R_{2} & \leq & I\left(Y_{2};W_{1}W_{2}Q\right)+I\left(Y_{1};W_{2}|W_{1}Q\right)\\ & & +I\left(Y_{1};U_{1}|W_{1}W_{2}Q\right)+2I\left(Y_{2};U_{2}|W_{1}W_{2}Q\right). \end{array}$$

Later, in [9], the authors showed that a simple HK scheme can achieve within one bit of the capacity of the two-user interference channel, considering three cases: (1) a weak interference channel, where ${\mathrm{INR}}_{1}<{\mathrm{SNR}}_{2}$ and ${\mathrm{INR}}_{2}<{\mathrm{SNR}}_{1}$; (2) a mixed interference channel, where ${\mathrm{INR}}_{1}\geq {\mathrm{SNR}}_{2}$ and ${\mathrm{INR}}_{2}<{\mathrm{SNR}}_{1}$ or ${\mathrm{INR}}_{1}<{\mathrm{SNR}}_{2}$ and ${\mathrm{INR}}_{2}\geq {\mathrm{SNR}}_{1}$; and (3) a strong interference channel, where ${\mathrm{INR}}_{1}\geq {\mathrm{SNR}}_{2}$ and ${\mathrm{INR}}_{2}\geq {\mathrm{SNR}}_{1}$. To complete this study, we present their results within one bit of the capacity rate region of the Gaussian interference channel for the weak interference channel. In Theorem 5 of [9], the authors proved that the achievable region $R\left(\min\left(1,I_{2}\right),\min\left(1,I_{1}\right)\right)$ is within one bit of the capacity region of the two-user weak Gaussian interference channel. For this, note that both the outer bound rate region and the HK rate region are delimited by straight lines of slopes 0,−1/2,−1,−2,∞, defined by the bounds $R_{1},R_{2},R_{1}+R_{2},2R_{1}+R_{2}$ and $R_{1}+2R_{2}$. In [9], this outer bound is given by (2)–(8). Those bounds are denoted by $UB_{R_{1}}$,$UB_{R_{2}}$,$UB_{R_{1}+R_{2}}$,$UB_{2R_{1}+R_{2}}$,$UB_{R_{1}+2R_{2}}$ and $HK_{R_{1}}$,$HK_{R_{2}}$,$HK_{R_{1}+R_{2}}$,$HK_{2R_{1}+R_{2}}$,$HK_{R_{1}+2R_{2}}$ for the outer bound and HK regions, respectively. The difference between these bounds is denoted by $\Delta_{R_{1}}=UB_{R_{1}}-HK_{R_{1}}$, $\Delta_{R_{2}}=UB_{R_{2}}-HK_{R_{2}}$, $\Delta_{R_{1}+R_{2}}=UB_{R_{1}+R2}-HK_{R_{1}+R_{2}}$, $\Delta_{2R_{1}+R_{2}}=UB_{2R_{1}+R2}-HK_{2R_{1}+R_{2}}$ and $\Delta_{R_{1}+2R_{2}}=UB_{R_{1}+2R2}-HK_{R_{1}+2R_{2}}$. Thus, the following condition is sufficient for the achievable region to be within 1 bit/s/Hz [9]:

$$\begin{array}{ccc} \Delta_{R_{1}} & < & 1\\ \Delta_{R_{2}} & < & 1\\ \Delta_{R_{1}+R_{2}} & < & 2\\ \Delta_{2R_{1}+R_{2}} & < & 3\\ \Delta_{R_{1}+2R_{2}} & < & 3 \end{array}$$

This is achieved by dividing the proof into four cases [9]:

(1) ${\mathrm{INR}}_{1}\geq 1$ and ${\mathrm{INR}}_{2}\geq 1$. In this case, ([9] [Corollary 1]) the achievable region $R\left(1,1\right)$ contains all the rate pairs $\left(R_{1},R_{2}\right)$, satisfying:
  (14) $$\begin{array}{ccc} R_{1} & \leq & \log\left(2+{\mathrm{SNR}}_{1}\right)-1 \end{array}$$
  (15) $$\begin{array}{ccc} R_{2} & \leq & \log\left(2+{\mathrm{SNR}}_{2}\right)-1 \end{array}$$
  (16) $$\begin{array}{ccc} R_{1}+R_{2} & \leq & \log\left({\mathrm{SNR}}_{2}+2{\mathrm{INR}}_{1}\right)+\log\left(1+\frac{{\mathrm{SNR}}_{1}+1}{{\mathrm{INR}}_{1}}\right)-2 \end{array}$$
  (17) $$\begin{array}{ccc} R_{1}+R_{2} & \leq & \log\left({\mathrm{SNR}}_{1}+2{\mathrm{INR}}_{2}\right)+\log\left(1+\frac{{\mathrm{SNR}}_{2}+1}{{\mathrm{INR}}_{2}}\right)-2 \end{array}$$
  (18) $$\begin{array}{ccc} R_{1}+R_{2} & \leq & \log\left(1+{\mathrm{INR}}_{1}+\frac{{\mathrm{SNR}}_{1}}{{\mathrm{INR}}_{2}}\right)+\log\left(1+{\mathrm{INR}}_{2}+\frac{{\mathrm{SNR}}_{2}}{{\mathrm{INR}}_{1}}\right)-2 \end{array}$$
  (19) $$\begin{array}{ccc} 2R_{1}+R_{2} & \leq & \log\left(1+{\mathrm{SNR}}_{1}+{\mathrm{INR}}_{1}\right)+\log\left(1+{\mathrm{INR}}_{2}+\frac{{\mathrm{SNR}}_{2}}{{\mathrm{INR}}_{1}}\right)\\ \; & \; & +\log\left(2+\frac{{\mathrm{SNR}}_{1}}{{\mathrm{INR}}_{2}}\right)-3 \end{array}$$
  (20) $$\begin{array}{ccc} R_{1}+2R_{2} & \leq & \log\left(1+{\mathrm{SNR}}_{2}+{\mathrm{INR}}_{2}\right)+\log\left(1+{\mathrm{INR}}_{1}+\frac{{\mathrm{SNR}}_{1}}{{\mathrm{INR}}_{2}}\right)\\ \; & \; & +\log\left(2+\frac{{\mathrm{SNR}}_{2}}{{\mathrm{INR}}_{1}}\right)-3. \end{array}$$
(2) ${\mathrm{INR}}_{1}<1$ and ${\mathrm{INR}}_{2}\geq 1$. In this case, the achievable region $R\left(1,{\mathrm{INR}}_{1}\right)$ contains all the rate pairs:
  (21) $$\begin{array}{ccc} R_{1} & \leq & \log\left(1+\frac{{\mathrm{SNR}}_{1}}{1+{\mathrm{INR}}_{1}}\right)-1 \end{array}$$
  (22) $$\begin{array}{ccc} R_{2} & \leq & \log\left(2+{\mathrm{SNR}}_{2}\right)-1 \end{array}$$
  (23) $$\begin{array}{ccc} R_{1}+R_{2} & \leq & \log\left({\mathrm{INR}}_{2}+\frac{{\mathrm{SNR}}_{1}}{1+{\mathrm{INR}}_{1}}\right)+\log\left(1+\frac{{\mathrm{SNR}}_{2}}{1+{\mathrm{INR}}_{2}}\right)-1 \end{array}$$
  (24) $$\begin{array}{ccc} R_{1}+R_{2} & \leq & \log\left(1+\frac{{\mathrm{SNR}}_{1}}{1+1{\mathrm{INR}}_{1}}\right)+\log\left(2+{\mathrm{SNR}}_{2}\right)-1 \end{array}$$
  (25) $$\begin{array}{ccc} R_{1}+R_{2} & \leq & \log\left({\mathrm{INR}}_{2}+\frac{{\mathrm{SNR}}_{1}}{1+{\mathrm{INR}}_{1}}\right)+\log\left(1+\frac{{\mathrm{SNR}}_{2}}{1+{\mathrm{INR}}_{2}}\right)-1 \end{array}$$
  (26) $$\begin{array}{ccc} 2R_{1}+R_{2} & \leq & \log\left(1+{\mathrm{SNR}}_{1}+{\mathrm{INR}}_{1}\right)+\log\left(1+{\mathrm{SNR}}_{2}+{\mathrm{INR}}_{2}\right)\\ \; & \; & +\log\left(1+{\mathrm{INR}}_{1}+\frac{{\mathrm{SNR}}_{1}}{{\mathrm{INR}}_{2}}\right)-\log2{\left(1+{\mathrm{INR}}_{1}\right)}^{2} \end{array}$$
  (27) $$\begin{array}{ccc} R_{1}+2R_{2} & \leq & \log\left(2+{\mathrm{SNR}}_{2}\right)+\log\left(1+{\mathrm{INR}}_{2}+\frac{{\mathrm{SNR}}_{1}}{1+{\mathrm{INR}}_{2}}\right)\\ \; & \; & +\log\left(1+\frac{1+{\mathrm{SNR}}_{2}}{{\mathrm{INR}}_{2}}\right)-2. \end{array}$$
(3) ${\mathrm{INR}}_{1}\geq 1$ and ${\mathrm{INR}}_{2}<1$. In this case, the achievable region $R\left({\mathrm{INR}}_{2},1\right)$ is similar to the one before.
(4) ${\mathrm{INR}}_{1}<1$ and ${\mathrm{INR}}_{2}<1$. In this case, the achievable region $R\left({\mathrm{INR}}_{2},{\mathrm{INR}}_{1}\right)$ contains only the following rate pairs:
  (28) $$\begin{array}{ccc} R_{1} & \leq & \log\left(1+\frac{{\mathrm{SNR}}_{1}}{1+{\mathrm{INR}}_{1}}\right) \end{array}$$
  (29) $$\begin{array}{ccc} R_{2} & \leq & \log\left(1+\frac{{\mathrm{SNR}}_{2}}{1+{\mathrm{INR}}_{2}}\right). \end{array}$$

The capacity is defined in [9] by $C({\mathrm{SNR}}_{1},{\mathrm{SNR}}_{2},{\mathrm{INR}}_{1},{\mathrm{INR}}_{2})$ with the parameters ${\mathrm{SNR}}_{1}$, ${\mathrm{SNR}}_{2}$, ${\mathrm{INR}}_{1}$ and ${\mathrm{INR}}_{2}$. Define the GDoF using [9] $D\left(\alpha_{1},\alpha_{2},\alpha_{3}\right)=\lim_{{\mathrm{SNR}}_{1},{\mathrm{SNR}}_{2},{\mathrm{INR}}_{1},{\mathrm{INR}}_{2}\to \infty }\;=\left\{\left(\frac{R_{1}}{{\mathrm{SNR}}_{1}},\frac{R_{2}}{{\mathrm{SNR}}_{2}}\right):\left(R_{1},R_{2}\right)\in C\left({\mathrm{SNR}}_{1},{\mathrm{SNR}}_{2},{\mathrm{INR}}_{1},{\mathrm{INR}}_{2}\right)\right\}$, where $\alpha_{1}=\frac{\log{\mathrm{SNR}}_{2}}{\log{\mathrm{SNR}}_{1}}$, $\alpha_{2}=\frac{\log{\mathrm{INR}}_{1}}{\log{\mathrm{SNR}}_{1}}$, $\alpha_{3}=\frac{\log{\mathrm{INR}}_{2}}{\log{\mathrm{SNR}}_{1}}$ and $R_{1}=d_{1}\log{\mathrm{SNR}}_{1}$, $R_{2}=d_{2}\log{\mathrm{SNR}}_{2}$ for $\left(d_{1},d_{2}\right)\in D$. Using various approximations, the GDoF for the weak interference channel is given by [9]:

(30) $$\begin{array}{ccc} d_{1} & \leq & 1 \end{array}$$

(31) $$\begin{array}{ccc} d_{2} & \leq & 1 \end{array}$$

(32) $$\begin{array}{ccc} d_{1}+\alpha_{1}d_{2} & \leq & \min\{1+{\left(\alpha_{1}-\alpha_{3}\right)}^{+},\alpha_{1}+{\left(1-\alpha_{2}\right)}^{+},\\ \; & \; & \max\left(\alpha_{2},1-\alpha_{3}\right)+\max\left(\alpha_{3},\alpha_{1}-\alpha_{2}\right)\} \end{array}$$

(33) $$\begin{array}{ccc} 2d_{1}+\alpha_{1}d_{2} & \leq & \max\left(1,\alpha_{2}\right)+\max\left(\alpha_{3},\alpha_{1}-\alpha_{2}\right)+\left(1-\alpha_{3}\right) \end{array}$$

(34) $$\begin{array}{ccc} d_{1}+2\alpha_{1}d_{2} & \leq & \max\left(\alpha_{1},\alpha_{3}\right)+\max\left(\alpha_{2},1-\alpha_{3}\right)+\left(\alpha_{1}-\alpha_{2}\right), \end{array}$$

### 2.2. Lattice Gaussian Coding

In this study, we use lattices due to their potential to align interference by means of lattice alignment for any number of users in an interference channel. Lattice codes also allow us to use higher dimensions, and some lattices are said to be AWGN-good if they are good for AWGN channels. We also note that the randomness of the codewords is useful, particularly when part of the codeword has to be treated as noise. Furthermore, the capacity to be within 1 Bit/s/Hz, as demonstrated in [9] and the GDoF, is based on Gaussian random codes. Due to this need, lattice Gaussian codes [19] are considered. In this section, we present the main results on this topic, which will be applied in the following sections for the interference channel.

**Definition 1**  (Lattice [22])**.** *A lattice is a regularly spaced array of points. It can be properly defined as: $\Lambda =\{x=\sum_{i=1}^{m}\lambda_{i}v_{i},\lambda_{i}\in Z\}$. The defined lattice has dimension m, where $v_{1},v_{2},\cdots ,v_{m}$ are linearly independent vectors in $R^{n}$ and $\left\{v_{1},v_{2},\cdots ,v_{m}\right\}$ is the basis of the lattice.*

**Definition 2**  (Theta series)**.** *Let $\Theta_{\Lambda }\left(q\right)$ be the theta series of a lattice* Λ*: $\Theta_{\Lambda }\left(q\right)=\sum_{\lambda \in \Lambda }q^{{∥\lambda ∥}^{2}}$, where, in this paper, $q=e^{-\frac{1}{2\sigma^{2}}}$.*

**Definition 3**  (AWGN-good [19])**.** *A sequence of lattices $\Lambda^{\left(n\right)}$ of increasing dimension n and volume-to-noise ratio (VNR), defined as $\gamma =\frac{V{\left(\Lambda \right)}^{\frac{2}{n}}}{\sigma^{2}}$, where $V\left(\Lambda \right)$ is the fundamental volume of the lattice* Λ*, is* AWGN-good *if, for all $P_{e}\in \left(0,1\right)$, $\lim_{n\to \infty }\gamma_{\Lambda^{\left(n\right)}}\left(\sigma \right)=2\pi e$ and if, for a fixed VNR greater than 2πe, $P_{e}$ vanishes in n, where $P_{e}=P\left\{W_{n}\notin V\left(\Lambda \right)\right\}$ is the error probability of minimum-distance lattice decoding, and where $\sigma^{2}$ is the power of the i.i.d Gaussian noise $W_{n}$.*

**Definition 4**  (Discrete Gaussian distribution [19])**.** *Define the* discrete Gaussian distribution *over* Λ *centered at $c\in R^{n}$ as the following discrete distribution taking values in λ∈Λ: $D_{\Lambda ,\sigma ,c}\left(\lambda \right)=\frac{f_{\sigma ,c}\left(\lambda \right)}{f_{\sigma ,c}\left(\Lambda \right)}\;\forall \lambda \in \Lambda$, where $f_{\sigma ,c}\left(\Lambda \right)≜\sum_{\lambda \in \Lambda }f_{\sigma ,c}\left(\lambda \right)$ and $f_{\sigma ,c}\left(x\right)$ is the Gaussian distribution of variance $\sigma^{2}$ centered at $c\in R^{n}$, $f_{\sigma ,c}\left(x\right)=\frac{1}{{\left(\sqrt{2\pi }\sigma \right)}^{n}}e^{-\frac{{∥x-c∥}^{2}}{2\sigma^{2}}}$. For convenience, $f_{\sigma }\left(x\right)=f_{\sigma ,0}\left(x\right)$ and $D_{\Lambda ,\sigma }=D_{\Lambda ,\sigma ,0}$.*

We also consider the Λ-periodic function:

$$\begin{array}{c} f_{\sigma ,\Lambda }\left(x\right)=\sum_{\lambda \in \Lambda }f_{\sigma ,\lambda }\left(x\right)=\frac{1}{{\left(\sqrt{2\pi }\sigma \right)}^{n}}\sum_{\lambda \in \Lambda }e^{-\frac{{∥x-\lambda ∥}^{2}}{2\sigma^{2}}}, \end{array}$$

for all $x\in R^{n}$.

**Definition 5**  (Discrete Gaussian distribution over a coset [19])**.** *The discrete Gaussian distribution over a coset of* Λ*; that is, the shifted lattice Λ−c is defined as $D_{\Lambda -c,\sigma }\left(\lambda -c\right)=\frac{f_{\sigma }\left(\lambda -c\right)}{f_{\sigma ,c}\left(\Lambda \right)}\;\forall \lambda \in \Lambda$. Note that $D_{\Lambda -c,\sigma }\left(\lambda -c\right)=D_{\Lambda ,\sigma ,c}\left(\lambda \right)$. Thus, they are a shifted version of each other.*

**Definition 6**  (Flatness factor [23])**.** *In [24], the notion of the flatness factor of a lattice* Λ *was introduced. An equivalent definition of the flatness factor is applied in [15,23]: $\epsilon_{\Lambda }\left(\sigma \right)≜{max}_{w\in R(\Lambda )}\left|V\left(\Lambda \right)f_{\sigma ,\Lambda }\left(w\right)-1\right|$. Thus, the ratio between $f_{\sigma ,\Lambda }\left(w\right)$ and the uniform distribution over $R\left(\Lambda \right)\subset R^{n}$ are within the range of $\left[1-\epsilon_{\Lambda }\left(\sigma \right),1+\epsilon_{\Lambda }\left(\sigma \right)\right]$, where R(Λ) is a fundamental region of the lattice* Λ. *The flatness factor of* Λ *is then given by [15]: $\epsilon_{\Lambda }\left(\sigma \right)={\left(\frac{\gamma }{2\pi }\right)}^{\frac{n}{2}}\Theta_{\Lambda }\left(e^{-\frac{1}{2\sigma^{2}}}\right)-1$.*

**Theorem 1**  ([19])**.** *∀σ and ∀δ, there exists a sequence of mod-p lattices $\Lambda^{\left(n\right)}\left(\sigma \right)$, such that*

(35) $$\begin{array}{c} \epsilon_{\Lambda^{\left(n\right)}}\leq \left(1+\delta \right)\cdot {\left(\frac{\gamma_{\Lambda^{\left(n\right)}}\left(\sigma \right)}{2\pi }\right)}^{\frac{n}{2}}; \end{array}$$

*that is, the flatness factor can exponentially reach zero for any fixed VNR $\gamma_{\Lambda^{\left(n\right)}}\left(\sigma \right)<2\pi$.*

From [25], mod-p lattices are defined as $\Lambda_{c}=pZ^{n}+C$, where *p* is a prime and *C* is a linear code over $Z_{p}$, which is the ring of integers modulo-*p*.

The following Lemma shows that when the flatness factor is small, the variance per dimension of the discrete Gaussian $D_{\Lambda ,\sigma ,c}$ is not so far from the one of the continuous Gaussian.

**Lemma 1**  ([15,19])**.** *Let x be sampled from the Gaussian distribution $D_{\Lambda ,\sigma ,c}$. If $\varepsilon ≜\epsilon_{\Lambda }\left(\sigma /\sqrt{\frac{\pi }{\pi -t}}\right)<1$ for 0<t<π, then*

(36) $$\begin{array}{ccc} |E\left[{∥x-c∥}^{2}\right]-n\sigma^{2}| & \leq & \frac{2\pi \varepsilon_{t}}{1-\varepsilon }\sigma^{2}. \end{array}$$

*where*

$$\varepsilon_{t}=\left\{\begin{array}{cc} \varepsilon , & \;t\geq 1/e\\ (t^{-4}+1)\varepsilon , & \;0<t<1/e \end{array}\right.$$

**Lemma 2**  (Entropy of discrete Gaussian [15])**.** *Let $x∼D_{\Lambda ,\sigma ,c}$. If $\varepsilon ≜\epsilon_{\Lambda }\left(\sigma /\sqrt{\frac{\pi }{\pi -t}}\right)<1$ for 0<t<π, then the entropy rate of x satisfies*

$$|\frac{1}{n}H\left(x\right)-\left[\log\left(\sqrt{2\pi e}\sigma \right)-\frac{1}{n}\log V(\Lambda )\right]|\leq \varepsilon^{\prime },$$

*where $\varepsilon^{\prime }=-\frac{\log(1-\varepsilon )}{n}+\frac{\pi \varepsilon_{t}}{n(1-\varepsilon )}$.*

The next lemma shows that the probability of a lattice Gaussian distribution falling outside of a ball of a radius larger than $\sqrt{n}\sigma$ is exponentially small.

**Lemma 3**  ([19])**.** *Let $x∼D_{\Lambda ,\sigma ,c}$ and $\varepsilon ≜\epsilon_{\Lambda }\left(\sigma \right)<1$. Then, for any ρ>1, the probability*

(37) $$P(∥x-c∥>\rho \cdot \sqrt{n}\sigma )\leq \frac{1+\varepsilon }{1-\varepsilon }\cdot e^{-nEsp(\rho^{2})}$$

*where $Esp\left(x\right)=\frac{1}{2}\left[x-1-\log(x)\right]$ for x>1 is the sphere packing exponent.*

**Definition 7**  (Semi-spherical noise [26])**.** *Let B(0,r) denote a ball of a radius r centered at zero. A sequence $Z_{n}$ is semi-spherical if ∀δ>0, $P\left(Z_{n}\in B\left(0,\left(1+\epsilon \right)\sqrt{n}\sigma \right)\right)>1-\delta$ for a sufficiently large n.*

Therefore, $x∼D_{\Lambda ,\sigma ,c}$ can be seen as semi-spherical noise. It is known that the sum of semi-spherical noise and AWGN is semi-spherical [26].

The following Lemma shows that if the flatness factor is small, the sum of the discrete Gaussian distribution and a continuous Gaussian distribution is very close to a continuous Gaussian distribution.

**Lemma 4**  ([19])**.** *Given any vector, $c\in R^{n}$ and $\sigma_{0},\sigma >0$. Let $\tilde{\sigma }=\frac{\sigma \sigma_{0}}{\sqrt{\sigma^{2}+\sigma_{0}^{2}}}$ and $\sigma_{s}^{\prime }=\sqrt{\sigma_{0}^{2}+\sigma^{2}}$. Consider the continuous distribution r on $R^{n}$ obtained by adding a continuous Gaussian of variance $\sigma^{2}$ to a discrete Gaussian $D_{\Lambda -c,\sigma_{0}}$:*

$$r\left(x\right)=\frac{1}{f_{\sigma_{0}}\left(\Lambda -c\right)}\sum_{t\in \Lambda -c}f_{\sigma_{0}}\left(t\right)f_{\sigma }\left(x-t\right),x\in R^{n}$$

*If $\varepsilon =\epsilon_{\Lambda }\left(\tilde{\sigma }\right)<\frac{1}{2}$, then $\frac{r(x)}{f_{\sigma_{s}^{\prime }}\left(x\right)}$ is uniformly close to 1:*

(38) $$\begin{array}{c} \forall x\in R^{n},\left|\frac{r(x)}{f_{\sigma_{s}^{\prime }}\left(x\right)}-1\right|\leq 4\varepsilon . \end{array}$$

As the distance between points is not uniform, the decoding is performed using MAP decoding. It is demonstrated in [19] that MAP decoding is equivalent to MMSE lattice decoding. The following lemma is given for the error performance of the AWGN-good lattices.

**Lemma 5**  ([19])**.** *If L is AWGN-good, the average error probability of the MAP decoder is bounded by*

(39) $$\begin{array}{c} P_{e}\leq \frac{1+\epsilon_{L}\left(\frac{\sigma_{0}^{2}}{\sqrt{\sigma_{0}^{2}+\sigma^{2}}}\right)}{1-\epsilon_{L}\left(\sigma_{0}\right)}e^{n_{L}E_{p}\left(\gamma_{L}\left(\tilde{\sigma }\right)\right)} \end{array}$$

*where $\tilde{\sigma }$ is defined in Lemma 4 and $E_{p}\left(\mu \right)$ denotes the Poltyrev exponent*

$$E_{p}\left(\mu \right)=\left\{\begin{array}{cc} \frac{1}{2}\left[(\mu -1)-\log\mu \right], & \;1<\mu \leq 2\\ \frac{1}{2}\log\frac{e\mu }{4}, & \;2\leq \mu \leq 4\\ \frac{\mu }{8}, & \;\mu \geq 4 \end{array}\right.$$

It is shown in [19] that in order to achieve this bound, the condition $\frac{\sigma_{0}^{2}}{\sigma^{2}}>e$ must be fulfilled; that is, the SNR is larger than *e*. Thus, the following theorem shows that by using a lattice Gaussian codebook, we can achieve a rate arbitrarily close to the channel capacity while making the error probability vanish exponentially, as long as SNR >e.

**Theorem 2**  ([19])**.** *Consider a lattice code whose codewords are drawn from the discrete Gaussian distribution $D_{L-c,\sigma_{s}}$ for an AWGN-good lattice. Assuming that $\varepsilon_{t}$ and ε are as defined in Lemma 1, ε′ is as defined in Lemma 2, and for some small $\varepsilon^{\prime \prime }\to 0$, if SNR >e, then any rate (as defined in [19])*

(40) $$R_{max}\geq \frac{1}{2}\log\left(1+\mathrm{SNR}\right)-\frac{\pi \varepsilon_{t}}{n_{L}\left(1-\varepsilon \right)}-\frac{1}{2}\varepsilon \prime \prime -\varepsilon \prime$$

*up to the channel capacity*

(41) $$\frac{1}{2}\log\left(1+\mathrm{SNR}\right)$$

*is achievable, while the error probability of MMSE lattice decoding vanishes exponentially fast as in (39).*

The development and proof of Theorem 2 can be found in [19].

## 3. Materials and Methods

### 3.1. Lattice Gaussian Coding for the Two-User Gaussian Interference Channel

In this section, we analyze the case for the two-user weak Gaussian interference channel using lattice Gaussian codes. Consider the following channel model:

(42) $$y_{i}=h_{ii}x_{i}+\sum_{j\neq i}^{2}h_{ji}x_{j}+z_{i}.$$

where $h_{ii}$ and $h_{ji}$ are the real direct and indirect channel gains, respectively; $x_{i}$ is the signal transmitted by transmitter *i*; $x_{j}$ is the signal transmitted by transmitter *j*; and $z_{i}$ is the additive white Gaussian noise with variance $\sigma^{2}$ and zero mean, i,j=1,2, i≠j. As in [3,9], the transmitted symbols are constructed using a common and a private message, given by $w_{i}$ and $u_{i}$, respectively, for user i=1,2. Thus, $x_{i}=w_{i}+u_{i}$. At receiver *i*, the common messages of both transmitters, $h_{ii}w_{i}$ and $h_{ji}w_{j}$ and the private desired message $h_{ii}u_{i}$ are decoded, while the interference private message $h_{ji}u_{j}$ is considered as noise, where j=1,2, j≠i. Define $S_{i}$ as the signal-to-noise ratio of user *i* and $I_{i}$ as the interference-to-noise ratio of user *i*. Furthermore, define, as presented in [9],

(43) $$\begin{array}{ccc} S_{ic} & ≜ & \frac{h_{ii}^{2}\sigma_{w_{i}}^{2}}{\sigma^{2}}, \end{array}$$

(44) $$\begin{array}{ccc} S_{ip} & ≜ & \frac{h_{ii}^{2}\sigma_{u_{i}}^{2}}{\sigma^{2}}, \end{array}$$

as the common and private signal-to-noise ratios of user *i*, respectively, and

(45) $$\begin{array}{ccc} I_{ic} & ≜ & \frac{h_{ji}^{2}\sigma_{w_{j}}^{2}}{\sigma^{2}}, \end{array}$$

(46) $$\begin{array}{ccc} I_{ip} & ≜ & \frac{h_{ji}^{2}\sigma_{u_{j}}^{2}}{\sigma^{2}}. \end{array}$$

as the common and private interference-to-noise ratios of user *i*, respectively. Thus, $S_{i}=S_{ic}+S_{ip}$ and $I_{i}=I_{ic}+I_{ip}$, considering the weak interference case, where $I_{i}<S_{j}$.

#### 3.1.1. Finding the Han–Kobayashi Rate Region with the Intersection of Two Two-User MACs

In [3], the best achievable rate region for a two-user interference channel was found using superposition coding. We will show that we can separate the problem into two multiple access channels (MACs), which can be intersected to obtain the achievable rate region obtained in [3].

**Figure 1:**
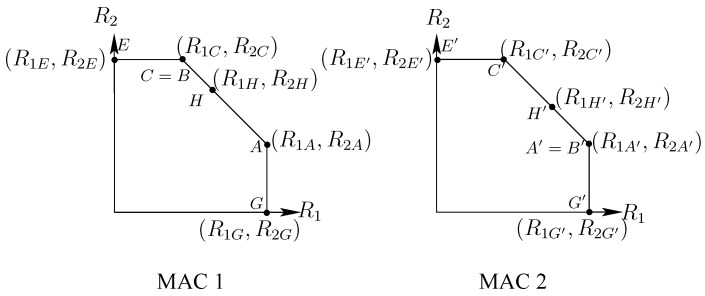
Representation of MAC 1 and MAC 2 rate regions.

**Lemma 6.**  *The extreme points for the achievable region of MAC 1 and MAC 2, respectively, are given by (see Figure 1):*

(47) $$\begin{array}{ccc} G & = & \left(I\left(Y_{1};W_{1}|W_{2}Q\right)+I\left(Y_{1};U_{1}|W_{1}W_{2}Q\right),0\right) \end{array}$$

(48) $$\begin{array}{ccc} A & = & \left(I\left(Y_{1};W_{1}|W_{2}Q\right)+I\left(Y_{1};U_{1}|W_{1}W_{2}Q\right),I\left(Y_{1};W_{2}Q\right)\right) \end{array}$$

(49) $$\begin{array}{ccc} E & = & \left(0,I\left(Y_{1};W_{2}|U_{1}W_{2}Q\right)\right) \end{array}$$

(50) $$\begin{array}{ccc} C & = & \left(I\left(Y_{1};W_{1}Q\right)+I\left(Y_{1};U_{1}|W_{1}Q\right),I\left(Y_{1};W_{2}|U_{1}W_{1}Q\right)\right) \end{array}$$

(51) $$\begin{array}{ccc} G\prime & = & \left(I\left(Y_{2};W_{1}|U_{2}W_{2}Q\right),0\right) \end{array}$$

(52) $$\begin{array}{ccc} A\prime & = & \left(I\left(Y_{2};W_{1}|U_{2}W_{2}Q\right),I\left(Y_{2};W_{2}\right)+I\left(Y_{2};U_{2}|W_{2}Q\right)\right) \end{array}$$

(53) $$\begin{array}{ccc} E\prime & = & \left(0,I\left(Y_{2};W_{2}|W_{1}Q\right)+I\left(Y_{2};U_{2}|W_{1}W_{2}Q\right)\right) \end{array}$$

(54) $$\begin{array}{ccc} C\prime & = & \left(I\left(Y_{2};W_{1}Q\right),I\left(Y_{2};W_{2}|W_{1}Q\right)+I\left(Y_{2};U_{2}|W_{1}W_{2}Q\right)\right) \end{array}$$

Proof: In order to find each of the MAC rate regions, we follow the procedure explained in [3], Appendix A. First, we notice that the MAC rate regions are delimited from above by only four straight lines, as opposed to the IC region, which is delimited by five. This is due to the fact that each MAC user only needs to decode both common messages and their own private messages. Thus, the only possible slopes for MAC 1 are given by 0,−1/2,−1,∞, and for MAC 2, they are given by 0,−1,−2,∞. Following the procedure explained in [3], Appendix A, it is straightforward to find (47)–(50). We found that point B is equal to C; therefore, we only have three slopes given by 0,−1,∞. It is also possible to find point *H*, where $R_{1C}+R_{2C}=R_{1H}+R_{2H}$.

(55) $$H=\left(I\left(Y_{1};W_{1}Q\right)+I\left(Y_{1};U_{1}|W_{1}W_{2}Q\right),I\left(Y_{1};W_{2}|W_{1}Q\right)\right)$$

We can follow a similar analysis for MAC 2, from (51) to (54). In this case, we find that point B’ is equal to A’; therefore, we have only three slopes as previously given by 0, −1, *∞*. It is also possible to find point $H^{\prime }$, where $R_{1A^{\prime }}+R_{2A^{\prime }}=R_{1H^{\prime }}+R_{2H^{\prime }}$.

(56) $$H^{\prime }=\left(I\left(Y_{2};W_{1}|W_{2}Q\right),I\left(Y_{2};W_{2}Q\right)+I\left(Y_{2};U_{2}|W_{1}W_{2}Q\right)\right)$$

**Lemma 7.**  *The achievable rate region found in ([3] [Theorem 4.1]) for a two-user interference channel can be found by intersecting the achievable rate region of two two-user multiple access channels, and this is given by:*

(57) $$\begin{array}{ccc} R_{1} & \leq & \underset{D_{1}}{\underset{︸}{I\left(Y_{1};U_{1}|W_{1}W_{2}Q\right)}}+\underset{T_{1}}{\underset{︸}{min\{I\left(Y_{1};W_{1}|W_{2}Q\right),I\left(Y_{2};W_{1}|U_{2}W_{2}Q\right)\}}} \end{array}$$

(58) $$\begin{array}{ccc} R_{2} & \leq & \underset{D_{2}}{\underset{︸}{I\left(Y_{2};U_{2}|W_{1}W_{2}Q\right)}}+\underset{T_{2}}{\underset{︸}{min\{I\left(Y_{2};W_{2}|W_{1}Q\right),I\left(Y_{1};W_{2}|U_{1}W_{1}Q\right)\}}} \end{array}$$

(59) $$\begin{array}{ccc} R_{1}+R_{2} & \leq & \underset{D_{1}}{\underset{︸}{I\left(Y_{1};U_{1}|W_{1}W_{2}Q\right)}}+\underset{D_{2}}{\underset{︸}{I\left(Y_{2};U_{2}|W_{1}W_{2}Q\right)}}+\underset{T_{1}+T_{2}}{\underset{︸}{I\left(Y_{1};W_{1}W_{2}Q\right)}} \end{array}$$

(60) $$\begin{array}{ccc} R_{1}+R_{2} & \leq & \underset{D_{1}}{\underset{︸}{I\left(Y_{1};U_{1}|W_{1}W_{2}Q\right)}}+\underset{D_{2}}{\underset{︸}{I\left(Y_{2};U_{2}|W_{1}W_{2}Q\right)}}+\underset{T_{1}+T_{2}}{\underset{︸}{I\left(Y_{2};W_{1}W_{2}Q\right)}} \end{array}$$

(61) $$\begin{array}{ccc} R_{1}+R_{2} & \leq & \underset{D_{1}}{\underset{︸}{I\left(Y_{1};U_{1}|W_{1}Q\right)}}+\underset{D_{2}}{\underset{︸}{I\left(Y_{2};U_{2}|W_{2}Q\right)}}\\ \; & \; & +\underset{T_{1}}{\underset{︸}{I\left(Y_{2};W_{1}|U_{2}W_{2}Q\right)}}+\underset{T_{2}}{\underset{︸}{I\left(Y_{1};W_{2}|U_{1}W_{1}Q\right)}} \end{array}$$

(62) $$\begin{array}{ccc} 2R_{1}+R_{2} & \leq & 2\underset{D_{1}}{\underset{︸}{I\left(Y_{1};U_{1}|W_{1}W_{2}Q\right)}}+\underset{D_{2}}{\underset{︸}{I\left(Y_{2};U_{2}|W_{2}Q\right)}}\\ \; & \; & +\underset{T_{1}}{\underset{︸}{I\left(Y_{2};W_{1}|U_{2}W_{2}Q\right)}}+\underset{T_{1}+T_{2}}{\underset{︸}{I\left(Y_{1};W_{1}W_{2}Q\right)}} \end{array}$$

(63) $$\begin{array}{ccc} R_{1}+2R_{2} & \leq & \underset{D_{1}}{\underset{︸}{I\left(Y_{1};U_{1}|W_{1}Q\right)}}+2\underset{D_{2}}{\underset{︸}{I\left(Y_{2};U_{2}|W_{1}W_{2}Q\right)}}\\ \; & \; & +\underset{T_{2}}{\underset{︸}{I\left(Y_{1};W_{2}|U_{1}W_{1}Q\right)}}+\underset{T_{1}+T_{2}}{\underset{︸}{I\left(Y_{2};W_{1}W_{2}Q\right)}} \end{array}$$

The proof of Lemma 7 can be found in Appendix A.

#### 3.1.2. Two-User Gaussian Interference Channel Using Lattice Gaussian Coding

**Figure 2:**
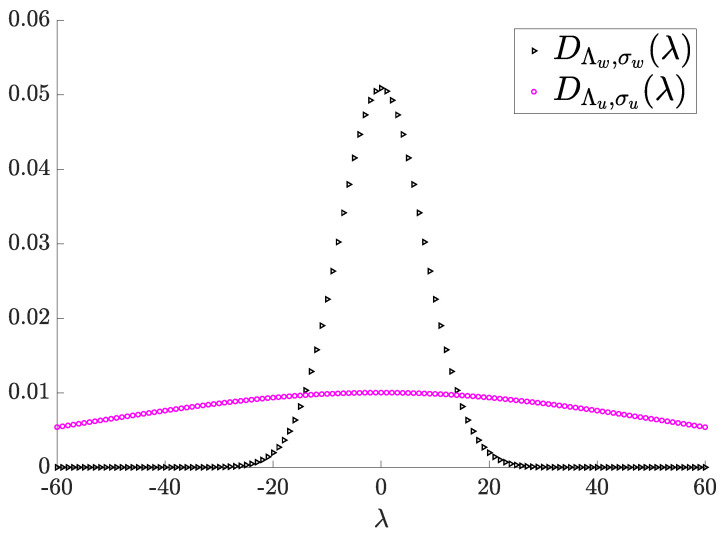
Representation of two superposed lattice Gaussians.

We assume that $h_{ji}w_{j}$ and $h_{ji}u_{j}$ for any i,j=1,2 are actually lattice codes and, more importantly, are in a lattice Gaussian distribution. Let us define the lattices properly in the lattice Gaussian distribution. We define that $h_{ii}w_{i}∼D_{\Delta_{i},\delta_{i}}$ where $\delta_{i}=h_{ii}\sigma_{w_{i}}$; $h_{ji}w_{j}∼D_{\Pi_{i},\rho_{i}}$ where $\rho_{i}=h_{ji}\sigma_{w_{j}}$; $h_{ii}u_{i}∼D_{\Gamma_{i},\gamma_{i}}$ where $\gamma_{i}=h_{ii}\sigma_{u_{i}}$; $h_{ji}u_{j}∼D_{\Psi_{i},\tau_{i}}$ where $\tau_{i}=h_{ji}\sigma_{u_{j}}$ and where $s∼D_{\Lambda ,\sigma }$ indicates that *s* distributes as the discrete lattice Gaussian distribution over Λ, centered in zero and with variance $\sigma^{2}$. Note that $x_{i}$ is the superposition of two lattice Gaussians. Figure 2 illustrates an example of a lattice Gaussian distribution of the private and common messages of $x_{i}$.

Based on the ideas of [3,9], at each receiver, both common and private desired messages must be decoded, along with the common interference message. However, the private interference message is considered noise. To decode similarly to [3], we consider successive decoding. Thus, while decoding one of the messages, the others are considered noise. This is not a problem when the codes used are Gaussian codes, as presented in [3,9]. Therefore, we not only work with lattice codes but with lattice Gaussian codes. The common messages are designed such that they are decodable at both receivers, while the private message must be designed such that it is decodable only at the desired receiver, and at the other receiver, it must be considered noise. Let us define the power of the private and common messages for transmitter *i* as $\sigma_{u_{i}}^{2}$ and $\sigma_{w_{i}}^{2}$, respectively, where i=1,2. From Lemma 4 it can be observed that if

(64) $$\epsilon_{\Psi_{i}}\left(\frac{\tau_{i}\sigma }{\sqrt{\tau_{i}^{2}+\sigma^{2}}}\right)=\epsilon_{\Psi_{i}}\left(\frac{h_{ji}\sigma_{u_{j}}\sigma }{\sqrt{h_{ji}^{2}\sigma_{u_{j}}^{2}+\sigma^{2}}}\right)<\frac{1}{2},$$

then $h_{ji}u_{j}+z_{i}$ is not far from a continuous distribution, and we can treat $h_{ji}u_{j}$ as noise. Thus, the new noise ${\tilde{z}}_{i}=h_{ji}u_{j}+z_{i}$ is an AWGN with variance $h_{ji}^{2}\sigma_{u_{j}}^{2}+\sigma^{2}$. Lemma 3 is applied to $h_{ii}w_{i}$, $h_{ji}w_{j}$ and $h_{ii}u_{i}$ with the flatness factor conditions:

(65) $$\begin{array}{ccc} \epsilon_{\Delta_{i}}\left(\delta_{i}\right) & < & 1 \end{array}$$

(66) $$\begin{array}{ccc} \epsilon_{\Pi_{i}}\left(\rho_{i}\right) & < & 1 \end{array}$$

(67) $$\begin{array}{ccc} \epsilon_{\Gamma_{i}}\left(\gamma_{i}\right) & < & 1, \end{array}$$

One important result of the separation of the problem on two MAC regions is the visualization of the decoding strategy. As in [3] the decoding strategy is the following. For MAC1, as can be observed from regions (47)–(50), we either decode $W_{2}$, then $W_{1}$ and finally $U_{1}$ or $W_{1}$, then $U_{1}$ and finally $W_{2}$, in both cases leaving the private interference message as noise. For MAC2, the approach is similar. From regions (51)–(54), we either decode $W_{2}$, then $U_{2}$ and finally $W_{1}$ or $W_{1}$, then $W_{2}$ and finally $U_{2}$. This can be formally expressed as follows. Considering a system given by (42), we will show the two possible ways of decoding at receiver *i*, i=1,2:

1. Decoding $h_{ii}w_{i}$, then $h_{ii}u_{i}$ and finally $h_{ji}w_{j}$: If we decode the desired common message first, $w_{i}$, to consider the rest of the messages as noise, we must apply Lemmas 4 and 3. Lemma 4 is applied to $h_{ji}u_{j}$, while Lemma 3 is applied to $h_{ji}w_{j}$ and $h_{ii}u_{i}$. Thus, we decode $w_{i}$ from $y_{k}=h_{ii}w_{i}+{\overset{ˇ}{z}}_{k}$, where ${\overset{ˇ}{z}}_{k}=h_{ji}w_{j}+h_{ii}u_{i}+h_{ji}u_{j}+z_{k}$ is the new semi-spherical noise. This is valid from Lemma 4 with the flatness factor condition
  (68) $$\epsilon_{\hat{\Psi_{i}}}\left(\frac{h_{ji}\sigma_{u_{j}}\sigma }{\sqrt{h_{ji}^{2}\sigma_{u_{j}}^{2}+\sigma^{2}}}\right)<\frac{1}{2}$$
  and from Lemma 3 with the flatness factor conditions
  (69) $$\epsilon_{\Pi_{i}}\left(\rho_{i}\right)<1$$
  and
  (70) $$\epsilon_{\Gamma_{i}}\left(\gamma_{i}\right)<1,$$
  Consider now Theorem 2. We have that
  (71) $$I\left(Y_{i};W_{i}\right)=\frac{1}{2}\log\left(1+\frac{h_{ii}^{2}\sigma_{w_{i}}^{2}}{h_{ji}^{2}\sigma_{w_{j}}^{2}+h_{ii}^{2}\sigma_{u_{i}}^{2}+h_{ji}^{2}\sigma_{u_{j}}^{2}+\sigma^{2}}\right)$$
  with the condition
  (72) $$\frac{h_{ii}^{2}\sigma_{w_{i}}^{2}}{h_{ji}^{2}\sigma_{w_{j}}^{2}+h_{ii}^{2}\sigma_{u_{i}}^{2}+h_{ji}^{2}\sigma_{u_{j}}^{2}+\sigma^{2}}>e$$
  Here, we decode the desired private message with a subset of the flatness factor conditions that were already defined in the first step. Thus, we decode $h_{ii}u_{i}$ from $y_{i}-h_{ii}{\hat{w}}_{i}=h_{ii}u_{i}+h_{ji}w_{j}+h_{ji}u_{j}+z_{i}$, where $\hat{w_{i}}$ is the estimated $w_{i}$, considering (64) and (66), which are the flatness factor conditions that make $h_{ji}u_{j}$ and $h_{ji}w_{j}$ part of the noise. Utilizing Theorem 2, we obtain
  (73) $$I\left(Y_{i};U_{i}|W_{i}\right)=\frac{1}{2}\log\left(1+\frac{h_{ii}^{2}\sigma_{u_{i}}^{2}}{h_{ji}^{2}\sigma_{w_{j}}^{2}+h_{ji}^{2}\sigma_{u_{j}}^{2}+\sigma^{2}}\right)$$
  where
  (74) $$\frac{h_{ii}^{2}\sigma_{u_{i}}^{2}}{h_{ji}^{2}\sigma_{w_{j}}^{2}+h_{ji}^{2}\sigma_{u_{j}}^{2}+\sigma^{2}}>e$$
  Finally, we can decode $w_{j}$ using $y_{k}-h_{ii}{\hat{w}}_{i}-h_{ii}{\hat{u}}_{i}=h_{ji}w_{j}+\left(h_{ji}u_{j}+z_{k}\right)$, where ${\hat{w}}_{i}$ and ${\hat{u}}_{i}$ are the estimated $w_{i}$ and $u_{i}$, respectively. Again, using Lemma 4, we can consider $h_{ji}u_{j}$ as part of the noise, with its respective flatness factor condition (64), and we can apply Theorem 2 to obtain
  (75) $$I\left(Y_{i};W_{j}|U_{i}W_{i}\right)=\frac{1}{2}\log\left(1+\frac{h_{ji}^{2}\sigma_{w_{j}}^{2}}{h_{ji}^{2}\sigma_{u_{j}}^{2}+\sigma^{2}}\right)$$
  where
  (76) $$\frac{h_{ji}^{2}\sigma_{w_{j}}^{2}}{h_{ji}^{2}\sigma_{u_{j}}^{2}+\sigma^{2}}>e$$
2. Decoding $h_{ji}w_{j}$, then $h_{ii}w_{i}$ and finally $h_{ii}u_{i}$:
  If we start by decoding the interference common message first, $h_{ji}w_{j}$, to consider the rest of the messages as noise, we apply Lemma 3 to $h_{ii}w_{i}$ and $h_{ii}u_{i}$ with the flatness factor conditions (65) and (67), and Lemma 4 to $h_{ji}u_{j}$ with the flatness factor condition (64).
  Then, using Theorem 2, we obtain
  (77) $$I\left(Y_{i};W_{j}\right)=\frac{1}{2}\log\left(1+\frac{h_{ji}^{2}\sigma_{w_{j}}^{2}}{h_{ii}^{2}\sigma_{w_{i}}^{2}+h_{ii}^{2}\sigma_{u_{i}}^{2}+h_{ji}^{2}\sigma_{u_{j}}^{2}+\sigma^{2}}\right)$$
  where
  (78) $$\frac{h_{ji}^{2}\sigma_{w_{j}}^{2}}{h_{ii}^{2}\sigma_{w_{i}}^{2}+h_{ii}^{2}\sigma_{u_{i}}^{2}+h_{ji}^{2}\sigma_{u_{j}}^{2}+\sigma^{2}}>e$$
  Here, we decode the desired common message $w_{i}$ from $y_{i}-h_{ji}{\hat{w}}_{j}=h_{ii}w_{i}+h_{ii}u_{i}+h_{ji}u_{j}+z_{i}$ again, where ${\hat{w}}_{j}$ is the estimated $w_{j}$, considering, as previously mentioned, $h_{ii}u_{i}$ and $h_{ji}u_{j}$ as noise with the conditions (67) and (64). Using Theorem 2, we obtain
  (79) $$I\left(Y_{i};W_{i}|W_{j}\right)=\frac{1}{2}\log\left(1+\frac{h_{ii}^{2}\sigma_{w_{i}}^{2}}{h_{ii}^{2}\sigma_{u_{i}}^{2}+h_{ji}^{2}\sigma_{u_{j}}^{2}+\sigma^{2}}\right)$$
  where
  (80) $$\begin{array}{c} \frac{h_{ii}^{2}\sigma_{w_{i}}^{2}}{h_{ii}^{2}\sigma_{u_{i}}^{2}+h_{ji}^{2}\sigma_{u_{j}}^{2}+\sigma^{2}}>e \end{array}$$
  Finally, once both common messages have been found, we can decode $u_{i}$ using $y_{k}-h_{ii}{\hat{w}}_{i}-h_{ji}{\hat{w}}_{j}=h_{ii}u_{i}+\left(h_{ji}u_{j}+z_{k}\right)$, where ${\hat{w}}_{i}$ and ${\hat{w}}_{j}$ are the estimated $w_{i}$ and $w_{j}$, respectively. Again using Lemma 4, we can consider $h_{ji}u_{j}$ as part of the noise, with its respective flatness factor condition (64), and we can apply Theorem 2 to obtain
  (81) $$I\left(Y_{i};U_{i}|W_{i}W_{j}\right)=\frac{1}{2}\log\left(1+\frac{h_{ii}^{2}\sigma_{u_{i}}^{2}}{h_{ji}^{2}\sigma_{u_{j}}^{2}+\sigma^{2}}\right)$$
  where
  (82) $$\frac{h_{ii}^{2}\sigma_{u_{i}}^{2}}{h_{ji}^{2}\sigma_{u_{j}}^{2}+\sigma^{2}}>e$$

### 3.2. Lattice Gaussian Coding for the K-User Interference Channel

In this section, we demonstrate how to use the previous results for the *K*-user interference channel utilizing lattice Gaussian coding.

Consider a *K*-user interference channel model given by:

(83) $$y_{i}=h_{ii}x_{i}+\sum_{i\neq j}h_{ji}x_{j}+z_{i},$$

where $h_{ii}$ and $h_{ji}$ are the real direct and indirect channel gains, respectively; $x_{i}$ is the signal transmitted by transmitter *i*; $x_{j}$ is the signal transmitted by transmitter *j*; and $z_{i}$ is the additive white Gaussian noise with variance $\sigma^{2}$ and zero mean, where i,j=1,⋯,K.

#### *K*-User Gaussian Interference Channel Using Lattice Gaussian Coding

The main idea of using lattice codes is to apply lattice alignment to the receivers so that we can ensure the model mimics a two-user interference channel.

**Lemma 8.**  *In a K-user Gaussian interference channel where lattice alignment is used, such that the channel resembles K two-user MACs, the number of lattice Gaussian codes needed to align interference is given by 2+4K.*

**Proof.**  To prove this let us begin with a three-user interference channel example. Our goal is to mimic the idea of the two-user interference channel where we can intersect the two-user MAC. For simplicity, let us consider the following channel model with only common messages:

(84) $$\begin{array}{ccc} y_{1} & = & h_{11}w_{1}+h_{21}w_{2}+h_{31}w_{3}+z_{1} \end{array}$$

(85) $$\begin{array}{ccc} y_{2} & = & h_{22}w_{2}+h_{12}w_{1}+h_{32}w_{3}+z_{2} \end{array}$$

(86) $$\begin{array}{ccc} y_{3} & = & h_{33}w_{3}+h_{13}w_{1}+h_{23}w_{2}+z_{3} \end{array}$$

In order to mimic a two-user interference channel, we will say that each user will see only one interference user in the following way:

For user *i*:

(87) $$\begin{array}{ccc} y_{i} & = & h_{ii}w_{i}+\sum_{j\neq i}h_{ji}w_{j}+z_{i} \end{array}$$

(88) $$\begin{array}{ccc} y_{int_{i}}=\sum_{j\neq i}y_{j} & = & \sum_{j\neq i}h_{jj}w_{j}+\sum_{j,l\neq i,l\neq j}h_{lj}w_{j}+\sum_{j\neq i}h_{ij}w_{i}\\ \; & + & \sum_{j\neq i}z_{j}. \end{array}$$

**Figure 3:**
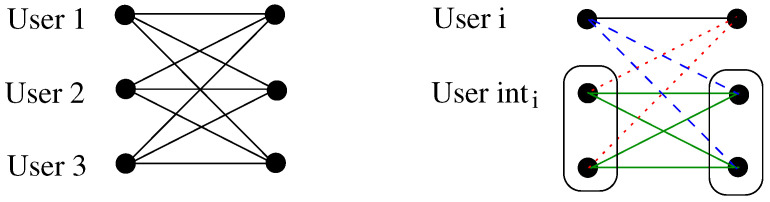
Representation of a three-user interference channel without (**left**) and with (**right**) the proposed alignment scheme, for i=1,2,3.

We have user *i* and the interference user, which is now the addition of two interferers; namely, user ${\mathrm{int}}_{i}$ (see Figure 3).

Assigning the lattices when K≥3 is more challenging than for the two-user case. Suppose we assign the lattices in the same way as the two-user interference channel. In this case, we would have that $h_{ii}w_{i}\in \Delta$, $\sum h_{ji}w_{j}\in \Theta$, $\sum h_{jl}w_{j}\in \Theta$, $\sum h_{ij}w_{i}\in \Theta$, for i,j=1,2,3 and i≠j. Then, we would have

(89) $$\begin{array}{ccc} y_{i} & \in & \Delta +\left(\Theta +\Theta \right) \end{array}$$

(90) $$\begin{array}{ccc} y_{int_{i}} & \in & \left(\Delta +\Delta +\Theta +\Theta \right)+\left(\Theta +\Theta \right). \end{array}$$

We can see it is not be possible to decode at ${\mathrm{int}}_{i}$ as we cannot decode $\sum_{j,l\neq i,l\neq j}h_{lj}w_{j}$ from $\sum_{j\neq i}h_{ij}w_{i}$. Let us consider the following strategy shown in Table 1.

In the first column, we show the perspective of each user. User 1 assigns $h_{ii}w_{i}\in \Delta$ for i=1,2,3, while it assigns $h_{j1}w_{j}\in \Pi$ and $h_{1j}w_{1}\in \Pi$ for j=2,3. User 2 assigns $h_{ii}w_{i}\in \Delta$ for i=1,2,3, while it assigns $h_{j2}w_{j}\in \Theta$ and $h_{2j}w_{2}\in \Theta$ for j=1,3. User 3 assigns $h_{ii}w_{i}\in \Delta$ for i=1,2,3, while it assigns $h_{j3}w_{j}\in \Upsilon$ and $h_{3j}w_{3}\in \Upsilon$ for j=1,2. The combination of all possible lattices for the case of $h_{ji}w_{j}$ for i,j=1,2,3, i≠j is given in the last line of Table 1. Note that, for example, the lattice ΠΘ is not necessarily a combination of lattices Π and Θ. It simply symbolizes a lattice that is useful for both $h_{21}w_{2}$ for users 1 and 2 and $h_{12}w_{1}$ for users 1 and 2. The same can be applied for $h_{31}w_{3}$ for users 1 and 3, $h_{13}w_{1}$ for users 1 and 3, $h_{23}w_{2}$ for users 2 and 3 and $h_{32}w_{3}$ for users 2 and 3. Thus, for each user, we obtain the following:

For user 1:

(91) $$\begin{array}{ccc} y_{1} & \in & \Delta +\left(\Pi \Theta +\Pi \Upsilon \right) \end{array}$$

(92) $$\begin{array}{ccc} y_{int_{1}} & \in & \left(\Delta +\Theta \Upsilon +\Theta \Upsilon +\Delta \right)+\left(\Pi \Theta +\Pi \Upsilon \right). \end{array}$$

Similarly, for user 2:

(93) $$\begin{array}{ccc} y_{2} & \in & \Delta +\left(\Pi \Theta +\Theta \Upsilon \right) \end{array}$$

(94) $$\begin{array}{ccc} y_{int_{2}} & \in & \left(\Delta +\Pi \Upsilon +\Pi \Upsilon +\Delta \right)+\left(\Pi \Theta +\Theta \Upsilon \right). \end{array}$$

Furthermore, for user 3:

(95) $$\begin{array}{ccc} y_{3} & \in & \Delta +\left(\Pi \Upsilon +\Theta \Upsilon \right) \end{array}$$

(96) $$\begin{array}{ccc} y_{int_{3}} & \in & \left(\Delta +\Pi \Theta +\Pi \Theta +\Delta \right)+\left(\Pi \Upsilon +\Theta \Upsilon \right). \end{array}$$

**Figure 4:**
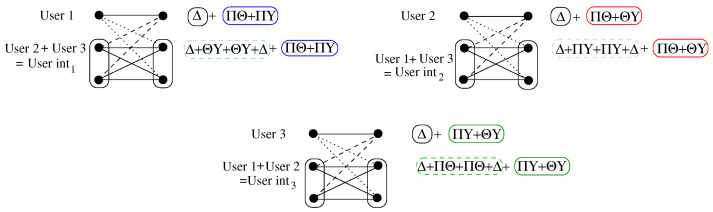
Lattice codes as seen by each receiver for the example described.

Here, let us focus on decoding. In order to obtain the same decoding rates at the desired receiver as in the previous two-user case, we need to be able to decode the following: common interferers, common desired messages and, finally, private desired messages or common desired messages, private desired messages and, finally, common interferers. For the interferer receiver, we need to be able to decode the following: common interferer messages, private interferer messages and, finally, common messages of user i or common messages of user i, common interferer messages and, finally, private interferer messages. In our three-user example without private messages, this means the following:

- At receiver 1, we decode to lattice Δ and then to lattice $\Pi_{1}$, where $\left(\Pi \Theta +\Pi \Upsilon \right)\subseteq \Pi_{1}$, or to lattice $\Pi_{1}$ and then to lattice Δ.
- At receiver ${\mathrm{int}}_{1}$, we decode to lattice $\Delta_{1}$, where $\left(\Delta +\Theta \Upsilon +\Theta \Upsilon +\Delta \right)\subseteq \Delta_{1}$, and then to $\Pi_{1}$ or first to $\Pi_{1}$ and then to $\Delta_{1}$.
  The process is similar for the other users. Thus, for our three-user interference channel example using only common messages, we need seven lattices to be able to decode three users and three interference users. This can be observed in Figure 4.

Following the same strategy, we find that for any K-user interference channel with common and private messages, we would need 2+4K lattices. Note that by using this strategy, there are lattices that repeat both at user *i* and user ${\mathrm{int}}_{i}$, thus allowing us to reduce the number of lattices that are needed to decode. □

The channel model is now given by:

(97) $$\begin{array}{ccc} y_{i} & = & h_{ii}w_{i}+\sum_{j\neq i}h_{ji}w_{j}+h_{ii}u_{i}+\sum_{j\neq i}h_{ji}u_{j}+z_{i} \end{array}$$

(98) $$\begin{array}{ccc} y_{int_{i}} & = & \sum_{j\neq i}h_{jl}w_{j}+\sum_{j\neq i}h_{jj}w_{j}+\sum_{j\neq i}h_{ij}w_{i}\\ \; & + & \sum_{j\neq i}h_{jl}u_{j}+\sum_{j\neq i}h_{jj}u_{j}+\sum_{j\neq i}h_{ij}u_{i}+z_{int_{i}}, \end{array}$$

where i,j,l=1,⋯,K. Let us properly define the lattices as follows: $h_{ii}w_{i}∼D_{\Delta ,\delta }$, where $\delta =h_{ii}\sigma_{w_{i}}$, $\sum_{j}h_{ji}w_{j}∼D_{\Pi_{i},\rho_{i}}$, $h_{ii}u_{i}∼D_{\Gamma ,\gamma }$ where $\gamma =h_{ii}\sigma_{u_{i}}$, $\sum_{j}h_{ji}u_{j}∼D_{\Psi_{i},\tau_{i}}$, $\sum_{j}h_{jl}w_{j}+h_{jj}w_{j}∼D_{\Lambda_{i},\lambda_{i}}$, where $\lambda_{i}=\sqrt{\left(\sum_{j\neq i}h_{jl}^{2}+\sum_{j\neq i}h_{jj}^{2}\right)\sigma_{w_{j}}^{2}}$, $\sum_{j}h_{ij}w_{i}∼D_{\Pi_{i},\rho_{i}}$, where $\rho_{i}=\sqrt{\sum_{j\neq i}h_{ji}^{2}\sigma_{w_{j}}^{2}}=\sqrt{\sum_{j\neq i}h_{ij}^{2}\sigma_{w_{i}}^{2}}$, $\sum_{j}h_{jl}u_{j}+h_{jj}u_{j}∼D_{\Upsilon_{i},\upsilon_{i}}$, where $\upsilon_{i}=\sqrt{\left(\sum_{j\neq i}h_{jl}^{2}+\sum_{j\neq i}h_{jj}^{2}\right)\sigma_{u_{j}}^{2}}$, $\sum_{j}h_{ij}u_{i}∼D_{\Psi_{i},\tau_{i}}$, where $\tau_{i}=\sqrt{\sum_{j\neq i}h_{ji}^{2}\sigma_{u_{j}}^{2}}=\sqrt{\sum_{j\neq i}h_{ij}^{2}\sigma_{u_{i}}^{2}}$, where i,j,l=1,⋯,K, j≠i and where we will assume that $\sigma_{w}=\sigma_{w_{i}}=\sigma_{w_{j}}$, $\sigma_{u}=\sigma_{u_{i}}=\sigma_{u_{j}}$ and $\sum h_{ji}^{2}=\sum h_{ij}^{2}$ for any i,j=1,⋯,K.

We can now define $S_{ic}≜\frac{h_{ii}^{2}\sigma_{w_{i}}^{2}}{\sigma^{2}}$, $S_{ip}≜\frac{h_{ii}^{2}\sigma_{u_{i}}^{2}}{\sigma^{2}}$, $I_{ic}≜\frac{\sum_{j\neq i}h_{ji}^{2}\sigma_{w_{j}}^{2}}{\sigma^{2}}$, $I_{ip}≜\frac{\sum_{j\neq i}h_{ji}^{2}\sigma_{u_{j}}^{2}}{\sigma^{2}}$, $S_{int_{ic}}≜\frac{\sum_{j\neq i}h_{jl}^{2}\sigma_{w_{j}}^{2}+\sum_{j\neq i}h_{jj}^{2}\sigma_{w_{j}}^{2}}{\sigma^{2}}$, $S_{int_{ip}}≜\frac{\sum_{j\neq i}h_{jl}^{2}\sigma_{u_{j}}^{2}+\sum_{j\neq i}h_{jj}^{2}\sigma_{u_{j}}^{2}}{\sigma^{2}}$, $I_{int_{ic}}≜\frac{\sum_{j\neq i}h_{ij}^{2}\sigma_{w_{i}}^{2}}{\sigma^{2}}$, $I_{int_{ip}}≜\frac{\sum_{j\neq i}h_{ij}^{2}\sigma_{u_{i}}^{2}}{\sigma^{2}}$.

From (97) and (98) for each i=1,⋯,K, we have two MAC regions with two possible rates, $R_{i}$ and $R_{int_{i}}$. Therefore, the interference channel rate region is given by:

(99) $$\begin{array}{ccc} R_{i} & \leq & \min\{I\left(Y_{i};W_{i}|W_{int_{i}}Q\right),I\left(Y_{int_{i}};W_{i}|U_{int_{i}}W_{int_{i}}Q\right)\}\\ \; & + & I\left(Y_{i};U_{i}|W_{i}W_{int_{i}}Q\right), \end{array}$$

(100) $$\begin{array}{ccc} R_{int_{i}} & \leq & \min\{I\left(Y_{int_{i}};W_{int_{i}}|W_{i}Q\right),I\left(Y_{i};W_{int_{i}}|U_{i}W_{i}Q\right)\}\\ \; & + & I\left(Y_{int_{i}};U_{int_{i}}|W_{i}W_{int_{i}}Q\right), \end{array}$$

(101) $$\begin{array}{ccc} R_{i}+R_{int_{i}} & \leq & \min\{I\left(Y_{i};W_{i}W_{int_{i}}Q\right),I\left(Y_{int_{i}};W_{i}W_{int_{i}}Q\right)\\ \; & , & I\left(Y_{i};W_{int_{i}}|W_{i}Q\right)+I\left(Y_{int_{i}};W_{i}|W_{int_{i}}Q\right)\}\\ \; & + & I\left(Y_{i};U_{i}|W_{i}W_{int_{i}}Q\right)+I\left(Y_{int_{i}};U_{int_{i}}|W_{i}W_{int_{i}}Q\right), \end{array}$$

(102) $$\begin{array}{ccc} 2R_{i}+R_{int_{i}} & \leq & I\left(Y_{i};W_{i}W_{int_{i}}Q\right)+I\left(Y_{int_{i}};W_{i}|W_{int_{i}}Q\right)\\ \; & + & 2I\left(Y_{i};U_{i}|W_{i}W_{int_{i}}Q\right)+I\left(Y_{int_{i}};U_{int_{i}}|W_{i}W_{int_{i}}Q\right) \end{array}$$

(103) $$\begin{array}{ccc} R_{i}+2R_{int_{i}} & \leq & I\left(Y_{int_{i}};W_{i}W_{int_{i}}Q\right)+I\left(Y_{i};W_{int_{i}}|W_{i}Q\right)\\ \; & + & I\left(Y_{i};U_{i}|W_{i}W_{int_{i}}Q\right)+2I\left(Y_{int_{i}};U_{int_{i}}|W_{i}W_{int_{i}}Q\right). \end{array}$$

for i=1,⋯,K,

In this case, Lemmas 3 and 4 can still be fulfilled using:

(104) $$\begin{array}{ccc} \epsilon_{\Psi_{i}}\left(\frac{\tau_{i}\sigma }{\sqrt{\tau_{i}^{2}+\sigma^{2}}}\right) & < & \frac{1}{2}, \end{array}$$

(105) $$\begin{array}{ccc} \epsilon_{\Delta }\left(\delta \right) & < & 1, \end{array}$$

(106) $$\begin{array}{ccc} \epsilon_{\Pi_{i}}\left(\rho_{i}\right) & < & 1, \end{array}$$

(107) $$\begin{array}{ccc} \epsilon_{\Lambda_{i}}\left(\lambda_{i}\right) & < & 1, \end{array}$$

(108) $$\begin{array}{ccc} \epsilon_{\Gamma }\left(\gamma \right) & < & 1, \end{array}$$

(109) $$\begin{array}{ccc} \epsilon_{\Upsilon_{i}}\left(\upsilon_{i}\right) & < & 1. \end{array}$$

As for the two-user case, we will consider decoding in the following manner. From (97) to (98):

1. Decoding at receiver *i*:
  (a) Decoding $h_{ii}w_{i}$, then $h_{ii}u_{i}$ and finally $\sum h_{ji}w_{j}$: If we decode the desired common message first, $h_{ii}w_{i}$, to consider the rest of the messages as noise, we have to apply Lemma 4 to $\sum h_{ji}u_{j}$ and Lemma 3 to $\sum h_{ji}w_{j}$ and $h_{ii}u_{i}$. Thus, we decode $w_{i}$ from $y_{i}=h_{ii}w_{i}+{\overset{ˇ}{z}}_{k}$, where ${\overset{ˇ}{z}}_{i}=\sum h_{ji}w_{j}+h_{ii}u_{i}+\sum h_{ji}u_{j}+z_{i}$ is the new semi-spherical noise. This is valid from Lemma 4 with the flatness factor condition
    (110) $$\epsilon_{\Psi_{i}}\left(\frac{\tau_{i}\sigma }{\sqrt{\tau_{i}^{2}+\sigma^{2}}}\right)<\frac{1}{2},$$
    and from Lemma 3 with the flatness factor conditions
    (111) $$\epsilon_{\Pi_{i}}\left(\rho_{i}\right)<1$$
    and
    (112) $$\epsilon_{\Gamma }\left(\gamma \right)<1,$$
    From Theorem 2, we have that
    (113) $$I\left(Y_{i};W_{i}\right)=\frac{1}{2}\log\left(1+\frac{h_{ii}^{2}\sigma_{w_{i}}^{2}}{\sum h_{ji}^{2}\sigma_{w_{j}}^{2}+h_{ii}^{2}\sigma_{u_{i}}^{2}+\sum h_{ji}^{2}\sigma_{u_{j}}^{2}+\sigma^{2}}\right)$$
    with the condition
    (114) $$\frac{h_{ii}^{2}\sigma_{w_{i}}^{2}}{\sum h_{ji}^{2}\sigma_{w_{j}}^{2}+h_{ii}^{2}\sigma_{u_{i}}^{2}+\sum h_{ji}^{2}\sigma_{u_{j}}^{2}+\sigma^{2}}>e$$
    We now decode the desired private message with a subset of the flatness factor conditions, which were already defined in the first step. Thus, we decode $h_{ii}u_{i}$ from $y_{i}-{\hat{w}}_{i}=h_{ii}u_{i}+\sum h_{ji}w_{j}+\sum h_{ji}u_{j}+z_{i}$, where $\hat{w_{i}}$ is the estimated $h_{ii}w_{i}$, considering (104) and (106), which are the flatness factor conditions that make $\sum h_{ji}u_{j}$ and $\sum h_{ji}w_{j}$ part of the noise. Utilizing Theorem 2, we obtain
    (115) $$I\left(Y_{i};U_{i}|W_{i}\right)=\frac{1}{2}\log\left(1+\frac{h_{ii}^{2}\sigma_{u_{i}}^{2}}{\sum h_{ji}^{2}\sigma_{w_{j}}^{2}+\sum h_{ji}^{2}\sigma_{u_{j}}^{2}+\sigma^{2}}\right)$$
    where
    (116) $$\frac{h_{ii}^{2}\sigma_{u_{i}}^{2}}{\sum h_{ji}^{2}\sigma_{w_{j}}^{2}+\sum h_{ji}^{2}\sigma_{u_{j}}^{2}+\sigma^{2}}>e$$
    Finally, we can decode $\sum h_{ji}w_{j}$ using $y_{k}-{\hat{w}}_{i}-{\hat{u}}_{i}=\sum h_{ji}w_{j}+\left(\sum h_{ji}u_{j}+z_{k}\right)$, where ${\hat{w}}_{i}$ and ${\hat{u}}_{i}$ are the estimated $h_{ii}w_{i}$ and $h_{ii}u_{i}$, respectively. Again, using Lemma 4, we can consider $\sum h_{ji}u_{j}$ as part of the noise, with its respective flatness factor condition (104), and we can apply Theorem 2 to obtain
    (117) $$I\left(Y_{i};W_{int_{i}}|U_{i}W_{i}\right)=\frac{1}{2}\log\left(1+\frac{\sum h_{ji}^{2}\sigma_{w_{j}}^{2}}{\sum h_{ji}^{2}\sigma_{u_{j}}^{2}+\sigma^{2}}\right)$$
    where
    (118) $$\frac{\sum h_{ji}^{2}\sigma_{w_{j}}^{2}}{\sum h_{ji}^{2}\sigma_{u_{j}}^{2}+\sigma^{2}}>e$$
  (b) Decoding $\sum h_{ji}w_{j}$, then $h_{ii}w_{i}$ and finally $h_{ii}u_{i}$:
    If we start by decoding the interference common message first, $\sum h_{ji}w_{j}$, to consider the rest of the messages as noise, we apply Lemma 3 to $h_{ii}w_{i}$ and $h_{ii}u_{i}$ with the flatness factor conditions (105) and (108) and Lemma 4 to $\sum h_{ji}u_{j}$ with the flatness factor conditions (104).
    Then, using Theorem 2, we obtain
    (119) $$I\left(Y_{i};W_{int_{i}}\right)=\frac{1}{2}\log\left(1+\frac{\sum h_{ji}^{2}\sigma_{w_{j}}^{2}}{h_{ii}^{2}\sigma_{w_{i}}^{2}+h_{ii}^{2}\sigma_{u_{i}}^{2}+\sum h_{ji}^{2}\sigma_{u_{j}}^{2}+\sigma^{2}}\right)$$
    where
    (120) $$\frac{\sum h_{ji}^{2}\sigma_{w_{j}}^{2}}{h_{ii}^{2}\sigma_{w_{i}}^{2}+h_{ii}^{2}\sigma_{u_{i}}^{2}+\sum h_{ji}^{2}\sigma_{u_{j}}^{2}+\sigma^{2}}>e$$
    Here, we decode the desired common message $h_{ii}w_{i}$ from $y_{i}-{\hat{w}}_{j}=h_{ii}w_{i}+h_{ii}u_{i}+\sum h_{ji}u_{j}+z_{i}$, where ${\hat{w}}_{j}$ is the estimated $\sum h_{ji}w_{j}$, considering, as previously mentioned, $h_{ii}u_{i}$ and $h_{ji}u_{j}$ as noise with the conditions (108) and (104). Using Theorem 2, we obtain
    (121) $$I\left(Y_{i};W_{i}|W_{int_{i}}\right)=\frac{1}{2}\log\left(1+\frac{h_{ii}^{2}\sigma_{w_{i}}^{2}}{h_{ii}^{2}\sigma_{u_{i}}^{2}+\sum h_{ji}^{2}\sigma_{u_{j}}^{2}+\sigma^{2}}\right)$$
    where
    (122) $$\frac{h_{ii}^{2}\sigma_{w_{i}}^{2}}{h_{ii}^{2}\sigma_{u_{i}}^{2}+\sum h_{ji}^{2}\sigma_{u_{j}}^{2}+\sigma^{2}}>e$$
    Finally, once both common messages have been found, we can decode $h_{ii}u_{i}$ using $y_{k}-{\hat{w}}_{i}-{\hat{w}}_{j}=h_{ii}u_{i}+\left(\sum h_{ji}u_{j}+z_{k}\right)$, where ${\hat{w}}_{i}$ and ${\hat{w}}_{j}$ are the estimated $h_{ii}w_{i}$ and $\sum h_{ji}w_{j}$, respectively. Again, using Lemma 4, we can consider $\sum h_{ji}u_{j}$ as part of the noise, with its respective flatness factor condition (104), and we can apply Theorem 2 to obtain
    (123) $$I\left(Y_{i};U_{i}|W_{i}W_{int_{i}}\right)=\frac{1}{2}\log\left(1+\frac{h_{ii}^{2}\sigma_{u_{i}}^{2}}{\sum h_{ji}^{2}\sigma_{u_{j}}^{2}+\sigma^{2}}\right)$$
    where
    (124) $$\frac{h_{ii}^{2}\sigma_{u_{i}}^{2}}{\sum h_{ji}^{2}\sigma_{u_{j}}^{2}+\sigma^{2}}>e$$
2. We will now decode at receiver $int_{i}$:
  (a) Decoding $\sum h_{jl}w_{j}+\sum h_{jj}w_{j}$, then $\sum h_{jl}u_{j}+\sum h_{jj}u_{j}$ and finally $\sum h_{ij}w_{i}$: If we decode the desired common message first, $\sum h_{jl}w_{j}+\sum h_{jj}w_{j}$, to consider the rest of the messages as noise, we must apply Lemmas 4 and 3. Lemma 4 is applied to $\sum h_{ij}u_{i}$, while Lemma 3 is applied to $\sum h_{jl}w_{j}+\sum h_{jj}w_{j}$ and $\sum h_{ij}w_{i}$. Thus, we decode $\sum h_{jl}w_{j}+\sum h_{jj}w_{j}$ from $y_{int_{i}}=\sum h_{jl}w_{j}+\sum h_{jj}w_{j}+{\overset{ˇ}{z}}_{k}$, where ${\overset{ˇ}{z}}_{i}=\sum h_{ij}w_{j}+\sum h_{jl}u_{j}+\sum h_{jj}u_{j}+\sum h_{ij}u_{i}+z_{int_{i}}$ is the new semi-spherical noise. This is valid from Lemma 4 with the flatness factor condition (104) and from Lemma 3 with the flatness factor conditions (109) and (106).
    Thus, from Theorem 2, we have that
    (125) $$I\left(Y_{int_{i}};W_{int_{i}}\right)=\frac{1}{2}\log\left(1+\frac{\sum h_{jl}^{2}\sigma_{w_{j}}^{2}+\sum h_{jj}^{2}\sigma_{w_{j}}^{2}}{\sum h_{ij}^{2}\sigma_{w_{i}}^{2}+\sum h_{jl}^{2}\sigma_{u_{j}}^{2}+\sum h_{jj}^{2}\sigma_{u_{j}}^{2}+\sum h_{ij}^{2}\sigma_{u_{i}}^{2}+\sigma^{2}}\right)$$
    with the condition
    (126) $$\frac{\sum h_{jl}^{2}\sigma_{w_{j}}^{2}+\sum h_{jj}^{2}\sigma_{w_{j}}^{2}}{\sum h_{ij}^{2}\sigma_{w_{i}}^{2}+\sum h_{jl}^{2}\sigma_{u_{j}}^{2}+\sum h_{jj}^{2}\sigma_{u_{j}}^{2}+\sum h_{ij}^{2}\sigma_{u_{i}}^{2}+\sigma^{2}}>e$$
    Here, we decode the desired private message with a subset of flatness factor conditions, which were already defined in the first step. Thus, we decode $\sum h_{jl}u_{j}+\sum h_{jj}u_{j}$ from $y_{int_{i}}-{\hat{w}}_{j}=\sum h_{jl}u_{j}+\sum h_{jj}u_{j}+\sum h_{ij}u_{i}+z_{int_{i}}$, where ${\hat{w}}_{j}$ is the estimated $\left(\sum h_{ji}+\sum h_{jj}\right)w_{j}$, considering (109) and (106), which are the flatness factor conditions that make $\sum h_{jl}u_{j}+\sum h_{jj}u_{j}$ and $\sum h_{ij}w_{i}$ part of the noise. Using Theorem 2, we obtain
    (127) $$I\left(Y_{int_{i}};U_{int_{i}}|W_{int_{i}}\right)=\frac{1}{2}\log\left(1+\frac{\sum h_{jl}^{2}\sigma_{u_{i}}^{2}+\sum h_{jj}^{2}\sigma_{u_{i}}^{2}}{\sum h_{ij}^{2}\sigma_{w_{i}}^{2}+\sum h_{ij}^{2}\sigma_{u_{i}}^{2}+\sigma^{2}}\right)$$
    where
    (128) $$\frac{\sum h_{jl}^{2}\sigma_{u_{i}}^{2}+\sum h_{jj}^{2}\sigma_{u_{i}}^{2}}{\sum h_{ij}^{2}\sigma_{w_{i}}^{2}+\sum h_{ij}^{2}\sigma_{u_{i}}^{2}+\sigma^{2}}>e$$
    Finally, we can decode $\sum h_{ij}w_{i}$ using $y_{int_{i}}-{\hat{w}}_{j}-{\hat{u}}_{j}=\sum h_{ij}w_{i}+\left(\sum h_{ij}u_{i}+z_{int_{i}}\right)$, where ${\hat{w}}_{j}$ and ${\hat{u}}_{j}$ are the estimated $\left(\sum h_{jl}+\sum h_{jj}\right)w_{j}$ and $\left(\sum h_{jl}+\sum h_{jj}\right)u_{j}$, respectively. Again, using Lemma 4, we can consider $\sum h_{ij}u_{i}$ as part of the noise, with its respective flatness factor condition (104), and we can apply Theorem 2 to obtain
    (129) $$I\left(Y_{int_{i}};W_{i}|U_{int_{i}}W_{int_{i}}\right)=\frac{1}{2}\log\left(1+\frac{\sum h_{ij}^{2}\sigma_{w_{i}}^{2}}{\sum h_{ij}^{2}\sigma_{u_{i}}^{2}+\sigma^{2}}\right)$$
    where
    (130) $$\frac{\sum h_{ij}^{2}\sigma_{w_{i}}^{2}}{\sum h_{ij}^{2}\sigma_{u_{i}}^{2}+\sigma^{2}}>e$$
  (b) Decoding $\sum h_{ij}w_{i}$, then $\sum h_{jl}w_{j}+\sum h_{jj}w_{j}$ and, finally, $\sum h_{jl}u_{j}+\sum h_{jj}u_{j}$:
    If we start by decoding the interference common message first, $\sum h_{ij}w_{i}$, to consider the rest of the messages as noise, we apply Lemma 3 to $\sum h_{jl}w_{j}+\sum h_{jj}w_{j}$ and $\sum h_{jl}u_{j}+\sum h_{jj}u_{j}$ with the flatness factor conditions (107) and (109) and Lemma 4 to $\sum h_{ij}u_{i}$ with the flatness factor conditions (104).
    Then, utilizing Theorem 2, we obtain
    (131) $$I\left(Y_{int_{i}};W_{i}\right)=\frac{1}{2}\log\left(1+\frac{\sum h_{ij}^{2}\sigma_{w_{i}}^{2}}{\sum h_{jl}^{2}\sigma_{w_{j}}^{2}+\sum h_{jj}^{2}\sigma_{w_{j}}^{2}+\sum h_{jl}^{2}\sigma_{u_{j}}^{2}+\sum h_{jj}^{2}\sigma_{u_{j}}^{2}+\sum h_{ij}^{2}\sigma_{u_{i}}^{2}+\sigma^{2}}\right)$$
    where
    (132) $$\frac{\sum h_{ij}^{2}\sigma_{w_{i}}^{2}}{\sum h_{jl}^{2}\sigma_{w_{j}}^{2}+\sum h_{jj}^{2}\sigma_{w_{j}}^{2}+\sum h_{jl}^{2}\sigma_{u_{j}}^{2}+\sum h_{jj}^{2}\sigma_{u_{j}}^{2}+\sum h_{ij}^{2}\sigma_{u_{i}}^{2}+\sigma^{2}}>e$$
    Here, we decode the desired common message $\sum h_{jl}w_{j}+\sum h_{jj}w_{j}$ from $y_{int_{i}}-{\hat{w}}_{i}=\sum h_{jl}w_{j}+\sum h_{jj}w_{j}+\sum h_{jl}u_{j}+\sum h_{jj}u_{j}+\sum h_{ij}u_{i}+z_{int_{i}}$ again, where ${\hat{w}}_{i}$ is the estimated $\sum h_{ij}w_{i}$, considering, as previously mentioned, $\sum h_{jl}u_{j}+\sum h_{jj}u_{j}$ and $\sum h_{ij}u_{i}$ as noise with the conditions (109) and (104). Using Theorem 2, we obtain
    (133) $$I\left(Y_{int_{i}};W_{int_{i}}|W_{i}\right)=\frac{1}{2}\log\left(1+\frac{\sum h_{jl}^{2}\sigma_{w_{j}}^{2}+\sum h_{jj}^{2}\sigma_{w_{j}}^{2}}{\sum h_{jl}^{2}\sigma_{u_{j}}^{2}+\sum h_{jj}^{2}\sigma_{u_{j}}^{2}+\sum h_{ij}^{2}\sigma_{u_{i}}^{2}+\sigma^{2}}\right)$$
    where
    (134) $$\frac{\sum h_{jl}^{2}\sigma_{w_{j}}^{2}+\sum h_{jj}^{2}\sigma_{w_{j}}^{2}}{\sum h_{jl}^{2}\sigma_{u_{j}}^{2}+\sum h_{jj}^{2}\sigma_{u_{j}}^{2}+\sum h_{ij}^{2}\sigma_{u_{i}}^{2}+\sigma^{2}}>e$$
    Finally, once both common messages have been found, we can decode $\sum h_{jl}u_{j}+\sum h_{jj}u_{j}$ by $y_{int_{i}}-{\hat{w}}_{i}-{\hat{w}}_{j}=\sum h_{jl}u_{j}+\sum h_{jj}u_{j}+\left(h_{ij}u_{i}+z_{int_{i}}\right)$, where ${\hat{w}}_{i}$ and ${\hat{w}}_{j}$ are the estimated $\sum h_{ij}w_{i}$ and $\left(\sum h_{jl}+\sum h_{jj}w_{j}\right)$, respectively. Again, using Lemma 4, we can consider $\sum h_{ij}u_{i}$ as part of the noise, with its respective flatness factor condition (104), and we can apply Theorem 2 to obtain
    (135) $$I\left(Y_{int_{i}};U_{int_{i}}|W_{i}W_{int_{i}}\right)=\frac{1}{2}\log\left(1+\frac{\sum h_{jl}^{2}\sigma_{u_{j}}^{2}+\sum h_{jj}^{2}\sigma_{u_{j}}^{2}}{\sum h_{ij}^{2}\sigma_{u_{i}}^{2}+\sigma^{2}}\right)$$
    where
    (136) $$\frac{\sum h_{jl}^{2}\sigma_{u_{j}}^{2}+\sum h_{jj}^{2}\sigma_{u_{j}}^{2}}{\sum h_{ij}^{2}\sigma_{u_{i}}^{2}+\sigma^{2}}>e$$

## 4. Results

Although some lemmas were obtained in Section 3, such as Lemmas 6–8, in this section, we will present the main results of this work.

### 4.1. The Power Constraints and GDoF of the Two-User Weak Gaussian Interference Channel with Lattice Gaussian Coding

Using the results from the previous section, we now find the power constraints for the private and common messages. These are stated in the next Lemma:

**Lemma 9.**  *For any type of interference, we have the following power constraints from (72), (74), (76), (78), (80) and (82),*

(137) $$\begin{array}{ccc} \sigma_{u_{i}}^{2} & > & \frac{\frac{\sigma^{2}e\left(e+1\right)}{h_{ii}^{2}}\left(\frac{e\left(e+1\right)h_{ji}^{2}}{h_{jj}^{2}}+1\right)}{1-\frac{h_{ij}^{2}h_{ji}^{2}}{h_{ii}^{2}h_{jj}^{2}}{\left(e+1\right)}^{2}e^{2}} \end{array}$$

(138) $$\begin{array}{ccc} \sigma_{w_{i}}^{2} & > & \max\{\frac{e{\left(e+1\right)}^{2}\left(h_{ij}^{2}\sigma_{u_{i}}^{2}+\sigma^{2}\right)}{h_{ij}^{2}}, \end{array}$$

(139) $$\begin{array}{ccc} \; & \; & \frac{e{\left(e+1\right)}^{2}\left(h_{ji}^{2}\sigma_{u_{j}}^{2}+\sigma^{2}\right)}{h_{ii}^{2}}\}, \end{array}$$

*for i,j=1,2, j≠i, and where we consider that $\frac{h_{11}^{2}h_{22}^{2}}{h_{12}^{2}h_{21}^{2}}>e^{2}{\left(e+1\right)}^{2}$.*

The proof of Lemma 9 can be found in Appendix B. Note that we choose $\frac{h_{11}^{2}h_{22}^{2}}{h_{12}^{2}h_{21}^{2}}>e^{2}{\left(e+1\right)}^{2}$, which does not contradict the weak interference scenario as, for weak interference, we need $\frac{h_{ii}^{2}}{h_{ij}^{2}}>1$. In order to fulfill the restrictions on the flatness factors, we can apply the same approach as in [19] where, for mod-p lattices, we can satisfy a small flatness factor if:

(140) $$\frac{V{\left(L\right)}^{2/n}}{2\pi \sigma_{s}^{2}}<1,$$

where we consider a discrete lattice Gaussian distribution over *L*, centered on zero and with variance $\sigma_{s}^{2}$. Then, for each of the defined lattices, to satisfy each of the flatness factor conditions, we must satisfy the following volume constraints:

(141) $$\begin{array}{ccc} V{\left(\Delta_{i}\right)}^{2/n} & < & 2\pi h_{ii}^{2}\sigma_{w_{i}}^{2}, \end{array}$$

(142) $$\begin{array}{ccc} V{\left(\Pi_{i}\right)}^{2/n} & < & 2\pi h_{ji}^{2}\sigma_{w_{j}}^{2}, \end{array}$$

(143) $$\begin{array}{ccc} V{\left(\Gamma_{i}\right)}^{2/n} & < & 2\pi h_{ii}^{2}\sigma_{u_{i}}^{2}, \end{array}$$

(144) $$\begin{array}{ccc} V{\left(\Psi_{i}\right)}^{2/n} & < & 2\pi \frac{h_{ji}^{2}\sigma_{u_{j}}^{2}\sigma^{2}}{h_{ji}^{2}\sigma_{u_{j}}^{2}+\sigma^{2}}, \end{array}$$

where we consider that the dimension *n* is the same for all lattices.

From (43)–(46), we can express the rates obtained in Section 3.1.1, Lemma 7 (equivalently (71), (73), (75), (77), (79) and (81)), with the following, where we have reduced the equations where possible,

(145) $$\begin{array}{ccc} R_{1} & \leq & \min\{\frac{1}{2}\log\left(1+\frac{S_{1c}}{S_{1p}+I_{1p}+1}\right),\frac{1}{2}\log\left(1+\frac{I_{2c}}{I_{2p}+1}\right)\}\\ \; & \; & +\frac{1}{2}\log\left(\frac{S_{1p}+I_{1p}+1}{I_{1p}+1}\right) \end{array}$$

(146) $$\begin{array}{ccc} R_{2} & \leq & \min\{\frac{1}{2}\log\left(1+\frac{S_{2c}}{S_{2p}+I_{2p}+1}\right),\frac{1}{2}\log\left(1+\frac{I_{1c}}{I_{1p}+1}\right)\}\\ \; & \; & +\frac{1}{2}\log\left(\frac{S_{2p}+I_{2p}+1}{I_{2p}+1}\right) \end{array}$$

(147) $$\begin{array}{ccc} R_{1}+R_{2} & \leq & \frac{1}{2}\log\left(\frac{S_{1}+I_{1}+1}{I_{1p}+1}\right)\left(\frac{I_{2p}+S_{2p}+1}{I_{2p}+1}\right) \end{array}$$

(148) $$\begin{array}{ccc} R_{1}+R_{2} & \leq & \frac{1}{2}\log\left(\frac{S_{2}+I_{2}+1}{I_{2p}+1}\right)\left(\frac{I_{1p}+S_{1p}+1}{I_{1p}+1}\right) \end{array}$$

(149) $$\begin{array}{ccc} R_{1}+R_{2} & \leq & \frac{1}{2}\log\left(\frac{S_{1p}+I_{1}+1}{I_{1p}+1}\right)\left(\frac{S_{2p}+I_{2}+1}{I_{2p}+1}\right) \end{array}$$

(150) $$\begin{array}{ccc} 2R_{1}+R_{2} & \leq & \frac{1}{2}\log\left(\frac{S_{1}+I_{1}+1}{S_{1p}+I_{1p}+1}\right)\left(\frac{S_{2p}+I_{2}+1}{I_{2p}+1}\right){\left(\frac{I_{1p}+S_{1p}+1}{I_{1p}+1}\right)}^{2} \end{array}$$

(151) $$\begin{array}{ccc} R_{1}+2R_{2} & \leq & \frac{1}{2}\log\left(\frac{S_{2}+I_{2}+1}{S_{2p}+I_{2p}+1}\right)\left(\frac{S_{1p}+I_{1}+1}{I_{1p}+1}\right){\left(\frac{I_{2p}+S_{2p}+1}{I_{2p}+1}\right)}^{2}. \end{array}$$

For the weak interference scenario $S_{1}>I_{2}$ and $S_{2}>I_{1}$. As in [9], the aim is to prove the constant gap and, ultimately, that we can obtain the same GDoF as in [9].

In [9], the HK region is used by $R\left(I_{1p},I_{2p}\right)$, where $I_{ip}$ is approximated by 1. The aim is to find the difference between the outer bound rate region and the HK rate region; in particular, a constant gap. In [9], the authors found that, in some cases, $I_{ip}=1$ is not enough to reduce the gap between the outer bound and the HK rate for $R_{1}$ and $R_{2}$; therefore, it is necessary to assign more power to the private interference. Thus, the achievable rate region is given by [9]$R\left(\min\left(1,I_{2}\right),\min\left(1,I_{1}\right)\right)$. This leads to four cases of reaching the constant gap. As in ([20] [Section 3.2]), we define $k_{i}=I_{jp}$, where $k_{i}$ can take values from 1 to $I_{j}$, and that $S_{ip}=\frac{S_{i}}{I_{j}}I_{jp}$. We obtain the following:

(152) $$\begin{array}{ccc} R_{1} & \leq & \min\{\frac{1}{2}\log\left(\frac{S_{1}+I_{1p}+1}{I_{1p}+1}\right),\frac{1}{2}\log\left(\frac{I_{2}+1}{I_{2p}+1}\right)\} \end{array}$$

(153) $$\begin{array}{ccc} \; & + & \frac{1}{2}\log\left(\frac{S_{1p}+I_{1p}+1}{I_{1p}+1}\right) \end{array}$$

(154) $$\begin{array}{ccc} \; & \leq & \frac{1}{2}\log\left(\frac{k_{2}+1+S_{1}}{k_{2}+1}\right) \end{array}$$

(155) $$\begin{array}{ccc} R_{2} & \leq & \frac{1}{2}\log\left(\frac{k_{1}+1+S_{2}}{k_{1}+1}\right) \end{array}$$

(156) $$\begin{array}{ccc} R_{1}+R_{2} & \leq & \frac{1}{2}\log\left(\frac{S_{1}+I_{1}+1}{I_{1}\left(k_{2}+1\right)}\right)+\frac{1}{2}\log\left(\frac{k_{2}S_{2}+k_{1}I_{1}+I_{1}}{k_{1}+1}\right) \end{array}$$

(157) $$\begin{array}{ccc} R_{1}+R_{2} & \leq & \frac{1}{2}\log\left(\frac{S_{2}+I_{2}+1}{I_{2}\left(k_{1}+1\right)}\right)+\frac{1}{2}\log\left(\frac{k_{1}S_{1}+k_{2}I_{2}+I_{2}}{k_{2}+1}\right) \end{array}$$

(158) $$\begin{array}{ccc} R_{1}+R_{2} & \leq & \frac{1}{2}\log\left(\frac{k_{1}\frac{S_{1}}{I_{2}}+I_{1}+1}{k_{2}+1}\right)+\frac{1}{2}\log\left(\frac{k_{2}\frac{S_{2}}{I_{1}}+I_{2}+1}{k_{1}+1}\right) \end{array}$$

(159) $$\begin{array}{ccc} 2R_{1}+R_{2} & \leq & \frac{1}{2}\log\left(S_{1}+I_{1}+1\right)+\frac{1}{2}\log\left(\frac{k_{2}\frac{S_{2}}{I_{1}}+I_{2}+1}{k_{1}+1}\right)+\frac{1}{2}\log\left(\frac{k_{1}S_{1}+k_{2}I_{2}+I_{2}}{I_{2}{\left(k_{2}+1\right)}^{2}}\right) \end{array}$$

(160) $$\begin{array}{ccc} R_{1}+2R_{2} & \leq & \frac{1}{2}\log\left(S_{2}+I_{2}+1\right)+\frac{1}{2}\log\left(\frac{k_{1}\frac{S_{1}}{I_{2}}+I_{1}+1}{k_{2}+1}\right)+\frac{1}{2}\log\left(\frac{k_{2}S_{2}+k_{1}I_{1}+I_{1}}{I_{1}{\left(k_{2}+1\right)}^{2}}\right), \end{array}$$

Let us define the difference between the outer bound rate region and the HK rate region, as presented in [9], as: $\Delta_{R_{1}}=UB_{R_{1}}-HK_{R_{1}}$, $\Delta_{R_{2}}=UB_{R_{2}}-HK_{R_{2}}$, $\Delta_{R_{1}+R_{2}}=UB_{R_{1}+R2}-HK_{R_{1}+R_{2}}$, $\Delta_{2R_{1}+R_{2}}=UB_{2R_{1}+R2}-HK_{2R_{1}+R_{2}}$ and $\Delta_{R_{1}+2R_{2}}=UB_{R_{1}+2R2}-HK_{R_{1}+2R_{2}}$. Let us focus on $\Delta_{R_{1}}$ and $\Delta_{R_{2}}$. Depending on the value of $k_{1}$ and $k_{2}$, the left or right part of the term inside the min in (154) or (155) is active. It was found in [9,20] that reassigning the value of $k_{i}$ and assigning more power to the private interference allows for the reduction in the gap between the outer bound and the HK rate for $R_{1}$ and $R_{2}$. In [9], the authors can also consider when $I_{i}$ < 1. In our case, we find that that is not possible since $I_{i}>1$ by construction. Thus, the lowest gap in $R_{1}$ and $R_{2}$, as presented in [9], is given by:

(161) $$\begin{array}{ccc} \Delta_{R_{1}} & < & \frac{1}{2}\log\left(k_{2}+1\right) \end{array}$$

(162) $$\begin{array}{ccc} \Delta_{R_{2}} & < & \frac{1}{2}\log\left(k_{1}+1\right) \end{array}$$

(163) $$\begin{array}{ccc} \Delta_{R_{1}+R_{2}} & < & \frac{1}{2}\log\left(\left(k_{1}+1\right)\left(k_{2}+1\right)\right) \end{array}$$

(164) $$\begin{array}{ccc} \Delta_{2R_{1}+R_{2}} & < & \frac{1}{2}\log\left({\left(k_{2}+1\right)}^{2}\left(k_{1}+1\right)\right) \end{array}$$

(165) $$\begin{array}{ccc} \Delta_{R_{1}+2R_{2}} & < & \frac{1}{2}\log\left({\left(k_{1}+1\right)}^{2}\left(k_{2}+1\right)\right). \end{array}$$

The above leads to the main Theorem of this section.

**Theorem 3.**  *The constant gap obtained in [9] for a two-user Gaussian interference channel using Gaussian codes is the same as that obtained using lattice Gaussian distribution when $\frac{h_{ii}^{2}}{h_{ij}^{2}}>2e\left(e+1\right)$ for i,j=1,2 and i≠j.*

The proof of Theorem 3 can be found in Appendix C.

It is then straightforward to obtain the GDoF, which is the same as in (30)–(34).

### 4.2. The Power Constraints and GDoF of the K-User Weak Gaussian Interference Channel with Lattice Gaussian Coding

Here, let us consider the case for the *K*-user Gaussian interference channel, as presented in Section 3. Assume that $\sigma_{w_{i}}=\sigma_{w}$ and $\sigma_{u_{i}}=\sigma_{u}$ for i,j,l=1,⋯,K, i≠j≠l. We have the following Lemma that shows the power constraints obtained for the private and common messages:

**Lemma 10.**  *For any type of interference, we have the following power constraints from (114), (116), (118), (120), (122), (124), (126), (128), (130), (132), (134) and (136):*

(166) $$\begin{array}{ccc} \sigma_{u}^{2}\; & > & \max\{\frac{\sigma^{2}e\left(e+1\right)}{h_{ii}^{2}-\sum h_{ji}^{2}e\left(e+1\right)}, \end{array}$$

(167) $$\begin{array}{ccc} \; & \; & \frac{\sigma^{2}eh_{ii}^{2}}{{\left(h_{ii}^{2}-e\sum h_{ji}^{2}\right)}^{2}-e\sum h_{ji}^{2}\left(h_{ii}^{2}+\sum h_{ji}^{2}\right)}, \end{array}$$

(168) $$\begin{array}{ccc} \; & \; & \frac{\sigma^{2}e\left(\sum h_{jl}^{2}+\sum h_{jj}^{2}\right)}{{\left(\sum h_{jl}^{2}+\sum h_{jj}^{2}-e\sum h_{ij}^{2}\right)}^{2}-e^{2}\sum h_{ij}^{2}\left(\sum h_{jl}^{2}+\sum h_{jj}^{2}+\sum h_{ij}^{2}\right)}, \end{array}$$

(169) $$\begin{array}{ccc} \; & \; & \frac{\sigma^{2}e\left(e+1\right)}{\left(\sum h_{jl}^{2}+\sum h_{jj}^{2}-e\left(e+1\right)\sum h_{ij}^{2}\right)}, \end{array}$$

(170) $$\begin{array}{ccc} \; & \; & \frac{\sigma^{2}e}{h_{ii}^{2}-e\sum h_{ji}^{2}}, \end{array}$$

(171) $$\begin{array}{ccc} \; & \; & \frac{\sigma^{2}e}{\sum h_{jl}^{2}+\sum h_{jj}^{2}-e\sum h_{ij}^{2}}\} \end{array}$$

(172) $$\begin{array}{ccc} \sigma_{w}^{2}\; & > & \max\{\frac{e\left(h_{ii}^{2}+\sum h_{ji}^{2}\right)\sigma_{u}^{2}+\sigma^{2}e}{h_{ii}^{2}-e\sum h_{ji}^{2}}, \end{array}$$

(173) $$\begin{array}{ccc} \; & \; & \frac{e\sum h_{ji}^{2}\sigma_{u}^{2}+\sigma^{2}e}{\sum h_{ji}^{2}}, \end{array}$$

(174) $$\begin{array}{ccc} \; & \; & \frac{e\left(\sum h_{jl}^{2}+\sum h_{jj}^{2}+\sum h_{ij}^{2}\right)\sigma_{u}^{2}+\sigma^{2}e}{\sum h_{jl}^{2}+\sum h_{jj}^{2}-e\sum h_{ij}^{2}}, \end{array}$$

(175) $$\begin{array}{ccc} \; & \; & \frac{e\sum h_{ij}^{2}\sigma_{u}^{2}+\sigma^{2}e}{\sum h_{ij}^{2}}, \end{array}$$

(176) $$\begin{array}{ccc} \; & \; & \frac{\sigma^{2}eh_{ii}^{2}}{\left(\sum h_{ij}^{2}-eh_{ii}^{2}\right)}\left(\frac{e+1}{h_{ii}^{2}-e\sum h_{ji}^{2}}\right), \end{array}$$

(177) $$\begin{array}{ccc} \; & \; & \frac{\sigma^{2}e\left(\sum h_{jl}^{2}+\sum h_{jj}^{2}\right)}{\left(\sum h_{ij}^{2}-e\left(\sum h_{jl}^{2}+\sum h_{jj}^{2}\right)\right)}\left(\frac{e+1}{\left(\sum h_{jl}^{2}+\sum h_{jj}^{2}\right)-e\sum h_{ji}^{2}}\right)\} \end{array}$$

*where $\frac{h_{ii}^{2}}{\sum h_{ji}^{2}}>e\left(e+1\right)$, ${\left(h_{ii}^{2}-e\sum h_{ji}^{2}\right)}^{2}>e\sum h_{ji}^{2}\left(h_{ii}^{2}+\sum h_{ji}^{2}\right)$, $\sum h_{jl}^{2}+\sum h_{jj}^{2}>e\left(e+1\right)\sum h_{ij}^{2}$, ${\left(\sum h_{jl}^{2}+\sum h_{jj}^{2}-e^{2}\sum h_{ij}^{2}\right)}^{2}>e^{2}\sum h_{ij}^{2}\left(\sum h_{jl}^{2}+\sum h_{jj}^{2}+\sum h_{ij}^{2}\right)$ and $\sum h_{ji}^{2}=\sum h_{ij}^{2}$, for i,j,l=1,⋯,K.*

The proof of Lemma 10 can be found in Appendix D.

As for the two-user interference channel, we must satisfy the following volume conditions for each lattice:

(178) $$\begin{array}{ccc} V{\left(\Delta \right)}^{2/n} & < & 2\pi h_{ii}^{2}\sigma_{w}^{2} \end{array}$$

(179) $$\begin{array}{ccc} V{\left(\Pi_{i}\right)}^{2/n} & < & 2\pi \sum h_{ji}^{2}\sigma_{w}^{2}=2\pi \sum h_{ij}^{2}\sigma_{w}^{2} \end{array}$$

(180) $$\begin{array}{ccc} V{\left(\Lambda_{i}\right)}^{2/n} & < & 2\pi \left(\sum h_{jl}^{2}+\sum h_{jj}^{2}\right)\sigma_{w}^{2} \end{array}$$

(181) $$\begin{array}{ccc} V{\left(\Gamma \right)}^{2/n} & < & 2\pi h_{ii}^{2}\sigma_{u}^{2} \end{array}$$

(182) $$\begin{array}{ccc} V{\left(\Upsilon_{i}\right)}^{2/n} & < & 2\pi \left(\sum h_{jl}^{2}+\sum h_{jj}^{2}\right)\sigma_{u}^{2} \end{array}$$

(183) $$\begin{array}{ccc} V{\left(\Psi_{i}\right)}^{2/n} & < & 2\pi \frac{\sum h_{ji}^{2}\sigma_{u}^{2}\sigma^{2}}{\sum h_{ji}^{2}\sigma_{u}^{2}+\sigma^{2}}=2\pi \frac{\sum h_{ij}^{2}\sigma_{u}^{2}\sigma^{2}}{\sum h_{ij}^{2}\sigma_{u}^{2}+\sigma^{2}}. \end{array}$$

As for the two-user case, we can formally express the *K*-user interference channel rates with alignment as follows:

(184) $$\begin{array}{ccc} R_{1} & \leq & \min\{\frac{1}{2}\log\left(1+\frac{S_{ic}}{S_{ip}+I_{ip}+1}\right),\frac{1}{2}\log\left(1+\frac{I_{int_{ic}}}{I_{int_{ip}}+1}\right)\}\\ \; & \; & +\frac{1}{2}\log\left(\frac{S_{ip}+I_{ip}+1}{I_{ip}+1}\right) \end{array}$$

(185) $$\begin{array}{ccc} R_{2} & \leq & \min\{\frac{1}{2}\log\left(1+\frac{S_{int_{ic}}}{S_{int_{ip}}+I_{int_{ip}}+1}\right),\frac{1}{2}\log\left(1+\frac{I_{ic}}{I_{ip}+1}\right)\}\\ \; & \; & +\frac{1}{2}\log\left(\frac{S_{int_{ip}}+I_{int_{ip}}+1}{I_{int_{ip}}+1}\right) \end{array}$$

(186) $$\begin{array}{ccc} R_{1}+R_{2} & \leq & \frac{1}{2}\log\left(\frac{S_{1}+I_{1}+1}{I_{ip}+1}\right)\left(\frac{I_{int_{ip}}+S_{int_{ip}}+1}{I_{int_{ip}}+1}\right) \end{array}$$

(187) $$\begin{array}{ccc} R_{1}+R_{2} & \leq & \frac{1}{2}\log\left(\frac{S_{2}+I_{2}+1}{I_{int_{ip}}+1}\right)\left(\frac{I_{ip}+S_{ip}+1}{I_{ip}+1}\right) \end{array}$$

(188) $$\begin{array}{ccc} R_{1}+R_{2} & \leq & \frac{1}{2}\log\left(\frac{S_{ip}+I_{1}+1}{I_{ip}+1}\right)\left(\frac{S_{int_{ip}}+I_{2}+1}{I_{int_{ip}}+1}\right) \end{array}$$

(189) $$\begin{array}{ccc} 2R_{1}+R_{2} & \leq & \frac{1}{2}\log\left(\frac{S_{1}+I_{1}+1}{S_{ip}+I_{ip}+1}\right)\left(\frac{S_{int_{ip}}+I_{2}+1}{I_{int_{ip}}+1}\right){\left(\frac{I_{ip}+S_{ip}+1}{I_{ip}+1}\right)}^{2} \end{array}$$

(190) $$\begin{array}{ccc} R_{1}+2R_{2} & \leq & \frac{1}{2}\log\left(\frac{S_{2}+I_{2}+1}{S_{int_{ip}}+I_{int_{ip}}+1}\right)\left(\frac{S_{ip}+I_{1}+1}{I_{ip}+1}\right){\left(\frac{I_{int_{ip}}+S_{int_{ip}}+1}{I_{int_{ip}}+1}\right)}^{2} \end{array}$$

We can observe that this is equivalent to the results obtained in (145)–(151) for the two-user case. Thus, the procedure is the same as the one for (161)–(165).

The main result of this section can be stated in the following theorem:

**Theorem 4.**  *The constant gap obtained in [9] for a two-user weak Gaussian interference channel using Gaussian codes is the same as that obtained for a K-user weak Gaussian interference channel using lattice Gaussian distribution when $\frac{h_{ii}^{2}}{\sum h_{ij}^{2}}>e\left(e+1\right)$, $\frac{\sum h_{jl}^{2}+\sum h_{jj}^{2}}{\sum h_{ij}^{2}}>e\left(e+1\right)$, where $\sum h_{ij}^{2}=\sum h_{ji}^{2}$.*

The proof of Theorem 4 can be found in Appendix E.

## 5. Discussion

In this section, we summarize and highlight the results of this research.

First, in Section 3, to understand the achievable rate of a two-user interference channel presented by [3], we divide the problem into two two-MAC regions. This allows us to visualize both the contribution of each user to the HK rate and the decoding order. Both of these are key for later designing the lattice Gaussian codes, particularly when extended to a *K*-user interference channel.

Second, in Section 3, in order to use the HK decoding method, we want to use lattice Gaussian distribution. For this, we define the lattices and the constraints for each of the lattice distributions. We begin with a two-user interference channel first, where we must consider Lemmas 3 and 4 to treat the common and private messages as noise in each step of the decoding process. Next, we apply Theorem 2 to each of the rates we found for the two-user interference channel, following the decoding order defined before.

From these, we demonstrate how to extend these results to the *K*-user interference channel, as explained in Section 3. We mimic a two-user interference channel using alignment, thus obtaining two users: *i* and $int_{i}$. The challenge is the strategy to choose the lattices to decode both at user *i* and user $int_{i}$. In addition, the strategy by which to choose the lattices (as shown in example Table 1) allows us to visualize that some lattices repeat for both user *i* and user $int_{i}$, allowing us to reduce the number of lattices used. We again verify Lemmas 3 and 4 and Theorem 2 to each of the rates obtained.

In Section 4, the main results are presented. First, for the two-user weak Gaussian interference channel, we obtain the common and private power constraints. From this, we verify that we can approximate $I_{ip}$ for i=1,2 to 1 if the condition $\frac{h_{ii}^{2}}{h_{ij}^{2}}>2e\left(e+1\right)$ holds (Theorem 3). Thus, this allows us to apply the same constraint as in [9], which leads to naturally obtaining the same constant gap and GDoF. We repeat the process for the *K*-user weak Gaussian interference channel, obtaining that we can also approximate $I_{ip}$ to 1 if the conditions $\frac{h_{ii}^{2}}{\sum h_{ij}^{2}}>e\left(e+1\right)$ and $\frac{\sum h_{jl}^{2}+\sum h_{jj}^{2}}{\sum h_{ij}^{2}}>e\left(e+1\right)$ hold (Theorem 4). Note that, in this case, the conditions are weaker than for the two-user interference channel, but we have an extra penalty, given by $\sum_{j\neq i}h_{ji}^{2}=\sum_{j\neq i}h_{ij}^{2}$.

## 6. Conclusions

In this paper, we presented a lattice Gaussian coding scheme for the *K*-user interference channel. We show that, through the use of random coding, we can obtain the same conditions that lead to the constant gap to the optimal rate and the GDoF for a two-user interference channel as obtained in [9]. Herein, we use the HK scheme with private and common messages and lattice Gaussian coding to obtain randomness within the structure of the lattice. We proved that we can obtain the conditions to find the same constant gap and GDoF as with random coding for the weak interference scenario. This was achieved by using various properties of the flatness factor of the lattices, with some constraints on the common and private message powers as well as the channel coefficients. We also show how this can be extended to a *K*-user weak Gaussian interference channel, as the interference can be aligned at the receivers using lattice Gaussian coding. 

## Figures and Tables

**Table 1:** Example of a three-user interference channel lattice assignment.

	$h_{11}w_{1}$	$h_{21}w_{2}$	$h_{31}w_{3}$	$h_{12}w_{1}$	$h_{22}w_{2}$	$h_{32}w_{3}$	$h_{13}w_{1}$	$h_{23}w_{2}$	$h_{33}w_{3}$
User 1	Δ	Π	Π	Π	Δ		Π		Δ
User 2	Δ	Θ		Θ	Δ	Θ		Θ	Δ
User 3	Δ		Υ		Δ	Υ	Υ	Υ	Δ
	Δ	ΠΘ	ΠΥ	ΠΘ	Δ	ΘΥ	ΠΥ	ΘΥ	Δ

## Data Availability

The original contributions presented in the study are included in the article, further inquiries can be directed to the corresponding author.

## Abbreviations

The following abbreviations are used in this manuscript:

SNR, S	Signal-to-noise ratio
DoF	Degrees of freedom
HK	Han and Kobayashi
GDoF	Generalized degrees of freedom
AWGN	Additive white Gaussian noise
INR, I	Interference-to-noise ratio
MAC	Multiple access channel
IC	Interference channel

## Appendix A. Proof of Lemma 7

The two MAC regions must be intersected to obtain the same rate region of the two-user interference channel as those in [3,20]. The strategy is as follows. Similar to [3], we define $R_{i}=D_{i}+T_{i}$, where $D_{i}$ represents the rate of the private message $U_{i}$, while $T_{i}$ represents the rate of the common message $W_{i}$. The rates of users 1 and 2 when the other user is not transmitting are trivial and given by:

(A1) $$R_{1}\leq \underset{D_{1}}{\underset{︸}{I\left(Y_{1};U_{1}|W_{1}W_{2}Q\right)}}+\underset{T_{1}}{\underset{︸}{\min\{I\left(Y_{1};W_{1}|W_{2}Q\right),I\left(Y_{2};W_{1}|U_{2}W_{2}Q\right)\}}}$$

(A2) $$R_{2}\leq \underset{D_{2}}{\underset{︸}{I\left(Y_{2};U_{2}|W_{1}W_{2}Q\right)}}+\underset{T_{2}}{\underset{︸}{\min\{I\left(Y_{2};W_{2}|W_{1}Q\right),I\left(Y_{1};W_{2}|U_{1}W_{1}Q\right)\}}}$$

For $R_{1}+R_{2}$, we have four possibilities:

• The contribution from MAC 1 and the contribution of the private message rate given by MAC 2
  (A3) $$R_{1}+R_{2}\leq \underset{D_{1}}{\underset{︸}{I\left(Y_{1};U_{1}|W_{1}W_{2}Q\right)}}+\underset{D_{2}}{\underset{︸}{I\left(Y_{2};U_{2}|W_{1}W_{2}Q\right)}}+\underset{T_{1}+T_{2}}{\underset{︸}{I\left(Y_{1};W_{1}W_{2}Q\right)}}$$
• The contribution from MAC 2 and the contribution of the private message rate given by MAC 1
  (A4) $$R_{1}+R_{2}\leq \underset{D_{1}}{\underset{︸}{I\left(Y_{1};U_{1}|W_{1}W_{2}Q\right)}}+\underset{D_{2}}{\underset{︸}{I\left(Y_{2};U_{2}|W_{1}W_{2}Q\right)}}+\underset{T_{1}+T_{2}}{\underset{︸}{I\left(Y_{2};W_{1}W_{2}Q\right)}}$$
• The contribution from the intersection of both MAC, where $R_{1A^{\prime }}<R_{1C}$ and $R_{2A^{\prime }}>R_{2C}$
  (A5) $$\begin{array}{ccc} R_{1}+R_{2} & \leq & \underset{D_{1}}{\underset{︸}{I\left(Y_{1};U_{1}|W_{1}Q\right)}}+\underset{D_{2}}{\underset{︸}{I\left(Y_{2};U_{2}|W_{2}Q\right)}}\\ \; & \; & +\underset{T_{1}}{\underset{︸}{I\left(Y_{2};W_{1}|U_{2}W_{2}Q\right)}}+\underset{T_{2}}{\underset{︸}{I\left(Y_{1};W_{2}|U_{1}W_{1}Q\right)}} \end{array}$$
• Finally, the contribution from the intersection of both MACs, where $R_{1A}<R_{1C^{\prime }}$ and $R_{2A}>R_{2C^{\prime }}$, is actually redundant and, therefore, discarded, as shown in [20].

Finally, we know that the HK rate region is also bounded by $2R_{1}+R_{2}$ and $R_{1}+2R_{2}$. This can be found using the following logic. For $2R_{1}+R_{2}$, we have two possibilities:

• The contribution of $R_{1}+R_{2}$ from MAC 1 and the contribution of $T_{1}$ and $D_{2}$ from MAC 2:
  (A6) $$\begin{array}{ccc} 2R_{1}+R_{2} & \leq & 2\underset{D_{1}}{\underset{︸}{I\left(Y_{1};U_{1}|W_{1}W_{2}Q\right)}}+\underset{D_{2}}{\underset{︸}{I\left(Y_{2};U_{2}|W_{2}Q\right)}}\\ \; & \; & +\underset{T_{1}}{\underset{︸}{I\left(Y_{2};W_{1}|U_{2}W_{2}Q\right)}}+\underset{T_{1}+T_{2}}{\underset{︸}{I\left(Y_{1};W_{1}W_{2}Q\right)}} \end{array}$$
• The contribution of $R_{1}+R_{2}$ from MAC 2 and the contribution of $T_{1}$ and $D_{1}$ from MAC 1, which is actually redundant and, therefore, discarded, as shown in [20]

For $R_{1}+2R_{2}$, we have two possibilities:

• The contribution of $R_{1}+R_{2}$ from MAC 1 and the contribution of $T_{2}$ and $D_{2}$ from MAC 2, which is actually redundant and, therefore, discarded, as shown in [20],
• The contribution of $R_{1}+R_{2}$ from MAC 2 and the contribution of $T_{2}$ and $D_{1}$ from MAC 1:
  (A7) $$\begin{array}{ccc} R_{1}+2R_{2} & \leq & \underset{D_{1}}{\underset{︸}{I\left(Y_{1};U_{1}|W_{1}Q\right)}}+2\underset{D_{2}}{\underset{︸}{I\left(Y_{2};U_{2}|W_{1}W_{2}Q\right)}}\\ \; & \; & +\underset{T_{2}}{\underset{︸}{I\left(Y_{1};W_{2}|U_{1}W_{1}Q\right)}}+\underset{T_{1}+T_{2}}{\underset{︸}{I\left(Y_{2};W_{1}W_{2}Q\right)}} \end{array}$$

## Appendix B. Proof of Lemma 9

At user *i*, if we first decode $w_{i}$, then $u_{i}$ and finally $w_{j}$, from (72), (74) and (76), we obtain the following power constraints:

(A8) $$\begin{array}{ccc} \sigma_{w_{j}}^{2} & > & \frac{e\left(h_{ji}^{2}\sigma_{u_{j}}^{2}+\sigma^{2}\right)}{h_{ji}^{2}} \end{array}$$

(A9) $$\begin{array}{ccc} \sigma_{u_{i}}^{2} & > & \frac{e\left(e+1\right)\left(h_{ji}^{2}\sigma_{u_{j}}^{2}+\sigma^{2}\right)}{h_{ii}^{2}} \end{array}$$

(A10) $$\begin{array}{ccc} \sigma_{w_{i}}^{2} & > & \frac{e{\left(e+1\right)}^{2}\left(h_{ji}^{2}\sigma_{u_{j}}^{2}+\sigma^{2}\right)}{h_{ii}^{2}} \end{array}$$

Similarly, at user *i*, if we first decode $w_{j}$, then $w_{i}$ and finally $u_{i}$, from (78), (80) and (82), we obtain the following power constraints:

(A11) $$\begin{array}{ccc} \sigma_{u_{i}}^{2} & > & \frac{e\left(h_{ji}^{2}\sigma_{u_{j}}^{2}+\sigma^{2}\right)}{h_{ii}^{2}} \end{array}$$

(A12) $$\begin{array}{ccc} \sigma_{w_{i}}^{2} & > & \frac{e\left(e+1\right)\left(h_{ji}^{2}\sigma_{u_{j}}^{2}+\sigma^{2}\right)}{h_{ii}^{2}} \end{array}$$

(A13) $$\begin{array}{ccc} \sigma_{w_{j}}^{2} & > & \frac{e{\left(e+1\right)}^{2}\left(h_{ji}^{2}\sigma_{u_{j}}^{2}+\sigma^{2}\right)}{h_{ji}^{2}} \end{array}$$

If we analyze each of the equations above assigning the values of i,j=1,2, we obtain twelve equations, six for user 1 and six for user 2. Then, we can consider four cases when decoding (i.e., at user 1, decode $w_{1}$, then $u_{1}$ and finally $w_{2}$, while at user 2, decode $w_{2}$, then $u_{2}$ and finally $w_{1}$, or at user 1 decode $w_{1}$, then $u_{1}$ and finally $w_{2}$, while at user 2, decode $w_{1}$, then $w_{2}$ and finally $u_{2}$ and so on). These cases are shown in Table A1.

**Table A1:** Common and private power messages obtained for each user, considering the decoding strategy presented in Section 3.1.2.

	User 1		User 2	
	$h_{11}w_{1}\to h_{11}u_{1}\to h_{21}w_{2}$	$h_{21}w_{2}\to h_{11}w_{1}\to h_{11}u_{1}$	$h_{22}w_{2}\to h_{22}u_{2}\to h_{12}w_{1}$	$h_{12}w_{1}\to h_{22}w_{2}\to h_{22}u_{2}$
$\sigma_{w_{1}}^{2}>$	$\frac{e{\left(e+1\right)}^{2}\left(h_{21}^{2}\sigma_{u_{2}}^{2}+\sigma^{2}\right)}{h_{11}^{2}}$	$\frac{e\left(e+1\right)\left(h_{21}^{2}\sigma_{u_{2}}^{2}+\sigma^{2}\right)}{h_{11}^{2}}$	$\frac{e\left(h_{12}^{2}\sigma_{u_{1}}^{2}+\sigma^{2}\right)}{h_{12}^{2}}$	$\frac{e{\left(e+1\right)}^{2}\left(h_{12}^{2}\sigma_{u_{1}}^{2}+\sigma^{2}\right)}{h_{12}^{2}}$
$\sigma_{w_{2}}^{2}>$	$\frac{e\left(h_{21}^{2}\sigma_{u_{2}}^{2}+\sigma^{2}\right)}{h_{21}^{2}}$	$\frac{e{\left(e+1\right)}^{2}\left(h_{21}^{2}\sigma_{u_{2}}^{2}+\sigma^{2}\right)}{h_{21}^{2}}$	$\frac{e{\left(e+1\right)}^{2}\left(h_{12}^{2}\sigma_{u_{1}}^{2}+\sigma^{2}\right)}{h_{22}^{2}}$	$\frac{e\left(e+1\right)\left(h_{12}^{2}\sigma_{u_{1}}^{2}+\sigma^{2}\right)}{h_{22}^{2}}$
$\sigma_{u_{1}}^{2}>$	$\frac{e\left(e+1\right)\left(h_{21}^{2}\sigma_{u_{2}}^{2}+\sigma^{2}\right)}{h_{11}^{2}}$	$\frac{e\left(h_{21}^{2}\sigma_{u_{2}}^{2}+\sigma^{2}\right)}{h_{11}^{2}}$		
$\sigma_{u_{2}}^{2}>$			$\frac{e\left(e+1\right)\left(h_{12}^{2}\sigma_{u_{1}}^{2}+\sigma^{2}\right)}{h_{22}^{2}}$	$\frac{e\left(h_{12}^{2}\sigma_{u_{1}}^{2}+\sigma^{2}\right)}{h_{22}^{2}}$

For each of these cases, we solve $\sigma_{u_{1}}^{2}$ and $\sigma_{u_{2}}^{2}$. For example, for the first case, we find:

(A14) $$\begin{array}{ccc} \sigma_{u_{1}}^{2} & > & \frac{e\left(e+1\right)\left(h_{21}^{2}\sigma_{u_{2}}^{2}+\sigma^{2}\right)}{h_{11}^{2}}, \end{array}$$

(A15) $$\begin{array}{ccc} \sigma_{u_{2}}^{2} & > & \frac{e\left(e+1\right)\left(h_{12}^{2}\sigma_{u_{1}}^{2}+\sigma^{2}\right)}{h_{22}^{2}} \end{array}$$

If we solve (A14) with (A15), we obtain:

(A16) $$\sigma_{u_{1}}^{2}>\frac{\frac{\sigma^{2}e\left(e+1\right)}{h_{11}^{2}}\left(\frac{e\left(e+1\right)h_{21}^{2}}{h_{22}^{2}}+1\right)}{1-\frac{h_{12}^{2}h_{21}^{2}}{h_{11}^{2}h_{22}^{2}}e^{2}{\left(e+1\right)}^{2}},$$

where we assume that $\frac{h_{11}^{2}h_{22}^{2}}{h_{12}^{2}h_{21}^{2}}>e^{2}{\left(e+1\right)}^{2}$.

If we repeat the previous reasoning for the four cases, we obtain four possible values for both $\sigma_{u_{1}}^{2}$ and $\sigma_{u_{2}}^{2}$. We choose the most restrictive one as the one presented in (137). Here, we take these values of $\sigma_{u_{1}}^{2}$ and $\sigma_{u_{2}}^{2}$, replace them in all the possible results of $\sigma_{w_{1}}^{2}$ and $\sigma_{w_{2}}^{2}$, and find the most restrictive ones, which are those presented in (139) and (138).

## Appendix C. Proof of Theorem 3

From (137) to (139), we have:

(A17) $$\begin{array}{ccc} I_{1p}=\frac{h_{21}^{2}}{\sigma^{2}}\sigma_{u_{2}}^{2} & > & \frac{h_{21}^{2}}{\sigma^{2}}\frac{\frac{\sigma^{2}e\left(e+1\right)}{h_{22}^{2}}\left(\frac{e\left(e+1\right)h_{12}^{2}}{h_{11}^{2}}+1\right)}{1-\frac{h_{21}^{2}h_{12}^{2}}{h_{22}^{2}h_{11}^{2}}{\left(e+1\right)}^{2}e^{2}}, \end{array}$$

(A18) $$\begin{array}{ccc} I_{1c}=\frac{h_{21}^{2}}{\sigma^{2}}\sigma_{w_{2}}^{2} & > & \max\{\frac{h_{21}^{2}}{\sigma^{2}}\frac{e{\left(e+1\right)}^{2}\left(h_{21}^{2}\sigma_{u_{2}}^{2}+\sigma^{2}\right)}{h_{21}^{2}}, \end{array}$$

(A19) $$\begin{array}{ccc} \; & \; & \frac{h_{21}^{2}}{\sigma^{2}}\frac{e{\left(e+1\right)}^{2}\left(h_{12}^{2}\sigma_{u_{1}}^{2}+\sigma^{2}\right)}{h_{22}^{2}}\}, \end{array}$$

(A20) $$\begin{array}{ccc} I_{2p}=\frac{h_{12}^{2}}{\sigma^{2}}\sigma_{u_{1}}^{2} & > & \frac{h_{12}^{2}}{\sigma^{2}}\frac{\frac{\sigma^{2}e\left(e+1\right)}{h_{11}^{2}}\left(\frac{e\left(e+1\right)h_{21}^{2}}{h_{22}^{2}}+1\right)}{1-\frac{h_{12}^{2}h_{21}^{2}}{h_{11}^{2}h_{22}^{2}}{\left(e+1\right)}^{2}e^{2}}, \end{array}$$

(A21) $$\begin{array}{ccc} I_{2c}=\frac{h_{12}^{2}}{\sigma^{2}}\sigma_{w_{1}}^{2} & > & \max\{\frac{h_{12}^{2}}{\sigma^{2}}\frac{e{\left(e+1\right)}^{2}\left(h_{12}^{2}\sigma_{u_{1}}^{2}+\sigma^{2}\right)}{h_{12}^{2}}, \end{array}$$

(A22) $$\begin{array}{ccc} \; & \; & \frac{h_{12}^{2}}{\sigma^{2}}\frac{e{\left(e+1\right)}^{2}\left(h_{21}^{2}\sigma_{u_{2}}^{2}+\sigma^{2}\right)}{h_{11}^{2}}\}, \end{array}$$

Define:

(A23) $$\begin{array}{c} \beta_{1}≜\frac{h_{11}^{2}}{h_{12}^{2}}\frac{1}{e\left(e+1\right)} \end{array}$$

(A24) $$\begin{array}{c} \beta_{2}≜\frac{h_{22}^{2}}{h_{21}^{2}}\frac{1}{e\left(e+1\right)} \end{array}$$

where $\beta_{1},\beta_{2}>1$. Thus, we have:

(A25) $$\begin{array}{ccc} I_{1p} & > & \frac{\beta_{1}+1}{\beta_{1}\beta_{2}-1}, \end{array}$$

(A26) $$\begin{array}{ccc} I_{1c} & > & \max\{e{\left(e+1\right)}^{2}\left(I_{1p}+1\right),\frac{\left(e+1\right)}{\beta_{2}}\left(I_{2p}+1\right)\} \end{array}$$

(A27) $$\begin{array}{ccc} I_{2p} & > & \frac{\beta_{2}+1}{\beta_{1}\beta_{2}-1}, \end{array}$$

(A28) $$\begin{array}{ccc} I_{2c} & > & \max\{e{\left(e+1\right)}^{2}\left(I_{2p}+1\right),\frac{\left(e+1\right)}{\beta_{1}}\left(I_{1p}+1\right)\} \end{array}$$

Consider what we obtained for $I_{1c}$ and $I_{2c}$ in (A26) and (A28). Observe that the left term in (A26) (or in (A28)) is always bigger than 1, as $I_{1p}>0$ and $I_{2p}>0$. Then, it is straightforward to find that I1 = I1c + I1p > 1 and I2 = I2c + I2p > 1. Then, we only need to analyze the constant gap for the case where $I_{1},I_{2}>1$ and $I_{1p}=I_{2p}=1$. If the first terms in $I_{1p}$ and $I_{2p}$ are bigger than $1>\frac{\beta_{1}+1}{\beta_{1}\beta_{2}-1}$ and $1>\frac{\beta_{2}+1}{\beta_{1}\beta_{2}-1}$, then

(A29) $$\begin{array}{ccc} \beta_{1} & > & 2 \end{array}$$

(A30) $$\begin{array}{ccc} \beta_{2} & > & 2. \end{array}$$

This completes the proof.

## Appendix D. Proof of Lemma 10

As $\sigma_{w_{i}}^{2}=\sigma_{w_{j}}^{2}=\sigma_{w}^{2}$ and $\sigma_{u_{i}}^{2}=\sigma_{u_{j}}^{2}=\sigma_{u}^{2}$, it is straightforward to find the following:

- Equations (172) and (173) from (114) and (118)
- Equations (174) and (175) from (126) and (130)
- Equation (176). From (120) and (122), we obtain
  (A31) $$\sigma_{w}^{2}>\max\left\{\frac{\left(h_{ii}^{2}+\sum h_{ji}^{2}\right)e\sigma_{u}^{2}+e\sigma^{2}}{\sum h_{ji}^{2}-h_{ii}^{2}e},\frac{\left(h_{ii}^{2}+\sum h_{ji}^{2}\right)e\sigma_{u}^{2}+e\sigma^{2}}{h_{ii}^{2}}\right\}$$
  From (124), we obtain:
  (A32) $$\sigma_{u}^{2}>\frac{e\sigma^{2}}{h_{ii}^{2}-e\sum h_{ji}^{2}}$$
  If we evaluate (A32) in (A31) and the maximum value, we obtain (176).
- Equation (177). From (132) and (134), we obtain
  (A33) $$\sigma_{w}^{2}>\max\left\{\frac{\left(\sum h_{jl}^{2}+\sum h_{jj}^{2}+\sum h_{ij}^{2}\right)e\sigma_{u}^{2}+e\sigma^{2}}{\sum h_{ij}^{2}-e\left(\sum h_{jl}^{2}+\sum h_{jj}^{2}\right)},\frac{\left(\sum h_{jl}^{2}+\sum h_{jj}^{2}+\sum h_{ij}^{2}\right)e\sigma_{u}^{2}+e\sigma^{2}}{\sum h_{jl}^{2}+\sum h_{jj}^{2}}\right\}$$
  From Equation (136), we obtain:
  (A34) $$\sigma_{u}^{2}>\frac{e\sigma^{2}}{\sum h_{jl}^{2}+\sum h_{jj}^{2}-e\sum h_{ij}^{2}}$$
  If we evaluate (A34) in (A33) and the maximum value, we obtain (177).

- Equations (166) and (167): From (116), we obtain:
  (A35) $$\sigma_{u}^{2}>\frac{e\sum h_{ji}^{2}\sigma_{w}^{2}+\sigma^{2}e}{\left(h_{ii}^{2}-\sum h_{ji}^{2}e\right)}$$
  This is evaluated by taking (173) to obtain (166) or (172) to obtain (167).
- Equations (168) and (169): From (128), we obtain:
  (A36) $$\sigma_{u}^{2}>\frac{e\sigma_{w}^{2}\sum h_{ji}^{2}+\sigma^{2}e}{\left(\sum h_{jl}^{2}+\sum h_{jj}^{2}\right)-e\sum h_{ij}^{2}}$$
  This is evaluated by taking (174) to obtain (168) and by taking (175) to obtain (169).
- Equation (170) can easily be derived from (124), while (171) can easily be derived from (136).

## Appendix E. Proof of Theorem 4

From (137) to (139), we have:

(A37) $$\begin{array}{ccc} I_{ip} & = & I_{int_{i}p}=\frac{\sum h_{ji}^{2}}{\sigma^{2}}\sigma_{u}^{2}, \end{array}$$

(A38) $$\begin{array}{ccc} \; & > & \max\{\frac{\sum h_{ji}^{2}}{\sigma^{2}}\frac{\sigma^{2}e\left(e+1\right)}{h_{ii}^{2}-\sum h_{ji}^{2}e\left(e+1\right)}, \end{array}$$

(A39) $$\begin{array}{ccc} \; & \; & \frac{\sum h_{ji}^{2}}{\sigma^{2}}\frac{\sigma^{2}eh_{ii}^{2}}{{\left(h_{ii}^{2}-e\sum h_{ji}^{2}\right)}^{2}-e\sum h_{ji}^{2}\left(h_{ii}^{2}+\sum h_{ji}^{2}\right)}, \end{array}$$

(A40) $$\begin{array}{ccc} \; & \; & \frac{\sum h_{ji}^{2}}{\sigma^{2}}\frac{\sigma^{2}e\left(\sum h_{jl}^{2}+\sum h_{jj}^{2}\right)}{{\left(\sum h_{jl}^{2}+\sum h_{jj}^{2}-e\sum h_{ij}^{2}\right)}^{2}-e^{2}\sum h_{ij}^{2}\left(\sum h_{jl}^{2}+\sum h_{jj}^{2}+\sum h_{ij}^{2}\right)}, \end{array}$$

(A41) $$\begin{array}{ccc} \; & \; & \frac{\sum h_{ji}^{2}}{\sigma^{2}}\frac{\sigma^{2}e\left(e+1\right)}{\left(\sum h_{jl}^{2}+\sum h_{jj}^{2}-e\left(e+1\right)\sum h_{ij}^{2}\right)}, \end{array}$$

(A42) $$\begin{array}{ccc} \; & \; & \frac{\sum h_{ji}^{2}}{\sigma^{2}}\frac{\sigma^{2}e}{h_{ii}^{2}-e\sum h_{ij}^{2}}, \end{array}$$

(A43) $$\begin{array}{ccc} \; & \; & \frac{\sum h_{ji}^{2}}{\sigma^{2}}\frac{\sigma^{2}e}{\sum h_{jl}^{2}+\sum h_{ll}^{2}-e\sum h_{ij}^{2}}\} \end{array}$$

(A44) $$\begin{array}{ccc} I_{ic} & = & I_{int_{i}c}=\frac{\sum h_{ji}^{2}}{\sigma^{2}}\sigma_{w}^{2}, \end{array}$$

(A45) $$\begin{array}{ccc} \; & > & \max\{\frac{\sum h_{ji}^{2}}{\sigma^{2}}\frac{e\left(h_{ii}^{2}+\sum h_{ji}^{2}\right)\sigma_{u}^{2}+\sigma^{2}e}{h_{ii}^{2}-e\sum h_{ji}^{2}}, \end{array}$$

(A46) $$\begin{array}{ccc} \; & \; & \frac{\sum h_{ji}^{2}}{\sigma^{2}}\frac{e\sum h_{ji}^{2}\sigma_{u}^{2}+\sigma^{2}e}{\sum h_{ji}^{2}}, \end{array}$$

(A47) $$\begin{array}{ccc} \; & \; & \frac{\sum h_{ji}^{2}}{\sigma^{2}}\frac{e\left(\sum h_{jl}^{2}+\sum h_{jj}^{2}+\sum h_{ij}^{2}\right)\sigma_{u}^{2}+\sigma^{2}e}{\sum h_{jl}^{2}+\sum h_{jj}^{2}-e\sum h_{ij}^{2}}, \end{array}$$

(A48) $$\begin{array}{ccc} \; & \; & \frac{\sum h_{ji}^{2}}{\sigma^{2}}\frac{e\sum h_{ij}^{2}\sigma_{u}^{2}+\sigma^{2}e}{\sum h_{ij}^{2}}, \end{array}$$

(A49) $$\begin{array}{ccc} \; & \; & \frac{\sum h_{ji}^{2}}{\sigma^{2}}\frac{\sigma^{2}eh_{ii}^{2}}{\left(\sum h_{ij}^{2}-eh_{ii}^{2}\right)}\left(\frac{e+1}{h_{ii}^{2}-e\sum h_{ji}^{2}}\right), \end{array}$$

(A50) $$\begin{array}{ccc} \; & \; & \frac{\sum h_{ji}^{2}}{\sigma^{2}}\frac{\sigma^{2}e\left(\sum h_{jl}^{2}+\sum h_{jj}^{2}\right)}{\left(\sum h_{ij}^{2}-e\left(\sum h_{jl}^{2}+\sum h_{jj}^{2}\right)\right)}\left(\frac{e+1}{\sum h_{jl}^{2}+\sum h_{jj}^{2}-e\sum h_{ji}^{2}}\right)\} \end{array}$$

We define the following:

(A51) $$\begin{array}{ccc} \tilde{\beta_{1}} & ≜ & \frac{h_{ii}^{2}}{\sum h_{ij}^{2}}\frac{1}{e\left(e+1\right)} \end{array}$$

(A52) $$\begin{array}{ccc} \tilde{\beta_{2}} & ≜ & \frac{\sum h_{jl}^{2}+\sum h_{jj}^{2}}{\sum h_{ji}^{2}}\frac{1}{e\left(e+1\right)}=\frac{\sum h_{jl}^{2}+\sum h_{jj}^{2}}{\sum h_{ij}^{2}}\frac{1}{e\left(e+1\right)}. \end{array}$$

where $\tilde{\beta_{1}},\tilde{\beta_{2}}>1$. Thus, we have:

(A53) $$\begin{array}{ccc} I_{ip}\; & > & \max\{\frac{1}{\tilde{\beta_{1}}-1}, \end{array}$$

(A54) $$\begin{array}{ccc} \; & \; & \frac{\tilde{\beta_{1}}e^{2}\left(e+1\right)}{{\left(\tilde{\beta_{1}}e\left(e+1\right)-e\right)}^{2}-e\left(\tilde{\beta_{1}}e\left(e+1\right)+1\right)}, \end{array}$$

(A55) $$\begin{array}{ccc} \; & \; & \frac{\tilde{\beta_{2}}e^{2}\left(e+1\right)}{{\left(\tilde{\beta_{2}}e\left(e+1\right)-e\right)}^{2}-e^{2}\left(\tilde{\beta_{2}}e\left(e+1\right)+1\right)}, \end{array}$$

(A56) $$\begin{array}{ccc} \; & \; & \frac{1}{\tilde{\beta_{2}}-1}, \end{array}$$

(A57) $$\begin{array}{ccc} \; & \; & \frac{1}{\tilde{\beta_{1}}\left(e+1\right)-1}, \end{array}$$

(A58) $$\begin{array}{ccc} \; & \; & \frac{1}{\tilde{\beta_{2}}\left(e+1\right)-1}\} \end{array}$$

(A59) $$\begin{array}{ccc} I_{ic} & > & \max\{\frac{e}{\tilde{\beta_{1}}e\left(e+1\right)-e}+\frac{\left(\tilde{\beta_{1}}e\left(e+1\right)+1\right)eI_{ip}}{\tilde{\beta_{1}}e\left(e+1\right)-e}, \end{array}$$

(A60) $$\begin{array}{ccc} \; & \; & e\left(1+I_{ip}\right), \end{array}$$

(A61) $$\begin{array}{ccc} \; & \; & \frac{e}{\tilde{\beta_{2}}e\left(e+1\right)-e}+\frac{\left(\tilde{\beta_{2}}e\left(e+1\right)+1\right)eI_{ip}}{\tilde{\beta_{2}}e\left(e+1\right)-e} \end{array}$$

(A62) $$\begin{array}{ccc} \; & \; & \frac{\tilde{\beta_{1}}e^{2}{\left(e+1\right)}^{2}}{\left(1-\tilde{\beta_{1}}e^{2}\left(e+1\right)\right)\left(\tilde{\beta_{1}}e\left(e+1\right)-e\right)}, \end{array}$$

(A63) $$\begin{array}{ccc} \; & \; & \frac{\tilde{\beta_{2}}e^{2}{\left(e+1\right)}^{2}}{\left(1-\tilde{\beta_{2}}e^{2}\left(e+1\right)\right)\left(\tilde{\beta_{2}}e\left(e+1\right)-e\right)}.\} \end{array}$$

In this case, we can verify that (A53) is bigger than (A54) and (A57); similarly, (A56) is bigger than (A55) and (A58), (A62) and (A63) are negative. Thus, we have:

(A64) $$I_{ip}>\max\left\{\frac{1}{\tilde{\beta_{1}}-1},\frac{1}{\tilde{\beta_{2}}-1}\right\}$$

(A65) $$I_{ic}>\max\left\{e\left(1+I_{ip}\right),\frac{\left(\tilde{\beta_{1}}e\left(e+1\right)+1\right)I_{ip}+1}{\tilde{\beta_{1}}\left(e+1\right)-1},\frac{\left(\tilde{\beta_{2}}e\left(e+1\right)+1\right)I_{ip}+1}{\tilde{\beta_{2}}\left(e+1\right)-1}\right\}$$

From $I_{ip}$ and $I_{ic}$, we see that it only suffices that $\tilde{\beta_{1}},\tilde{\beta_{2}}>\frac{1}{e+1}$, which is already fulfilled as $\tilde{\beta_{1}},\tilde{\beta_{2}}>1$. This completes the proof.
